# Impact of the spatial distribution of positive charges on the catalytic activity of NiSOD bioinspired complexes

**DOI:** 10.1007/s00775-026-02150-3

**Published:** 2026-05-19

**Authors:** Pawel Guinard, Jacques Pécaut, Alan Le-Goff, Carole Duboc, Pascale Delangle, Sarah Hostachy

**Affiliations:** 1 Univ. Grenoble Alpes, CEA, CNRS, Grenoble INP, IRIG, SyMMES, 38000 Grenoble, France; 2 Univ. Grenoble Alpes, CNRS, DCM, 38000 Grenoble, France

**Keywords:** Nickel superoxide dismutase, Metallopeptide, ATCUN motif, Biomimetic complexes

## Abstract

In order to mitigate superoxide-induced damage, cells rely on superoxide dismutases (SODs), a family of metalloenzymes that catalyze the dismutation of superoxide. These enzymes display a positively-charged substrate entrance channel for superoxide guidance to the active metal center. ATCUN-like Ni(II) complexes have been shown to mimic NiSOD effectively and benefit from versatile peptide structures, which are ideal for introducing positive charges through arginine residues at various positions to modulate catalytic properties. We describe here five novel ATCUN-like Ni(II) complexes, with arginines at various positions in close proximity to the metal center. All the peptides form monometallic square planar Ni(II) complexes in water at pH 7.4, with either a N_2_S_2_ coordination (*S*-series) or an equilibrium between N_2_S_2_ and N_3_S_1_ coordination modes (*N/S*-series). The *S*-series demonstrates a clear beneficial effect of the charge increase from − 2 to + 2 on the pseudo-first order catalytic constant. Furthermore, the data obtained for the *N/S* complexes demonstrate that introducing an arginine residue in the central position of the ATCUN-like motif does not enhance activity, but actually impedes catalysis. These findings show that both the distance of the charged residues from the metal center and their positioning in space are of prime importance.

**Graphical abstract:**

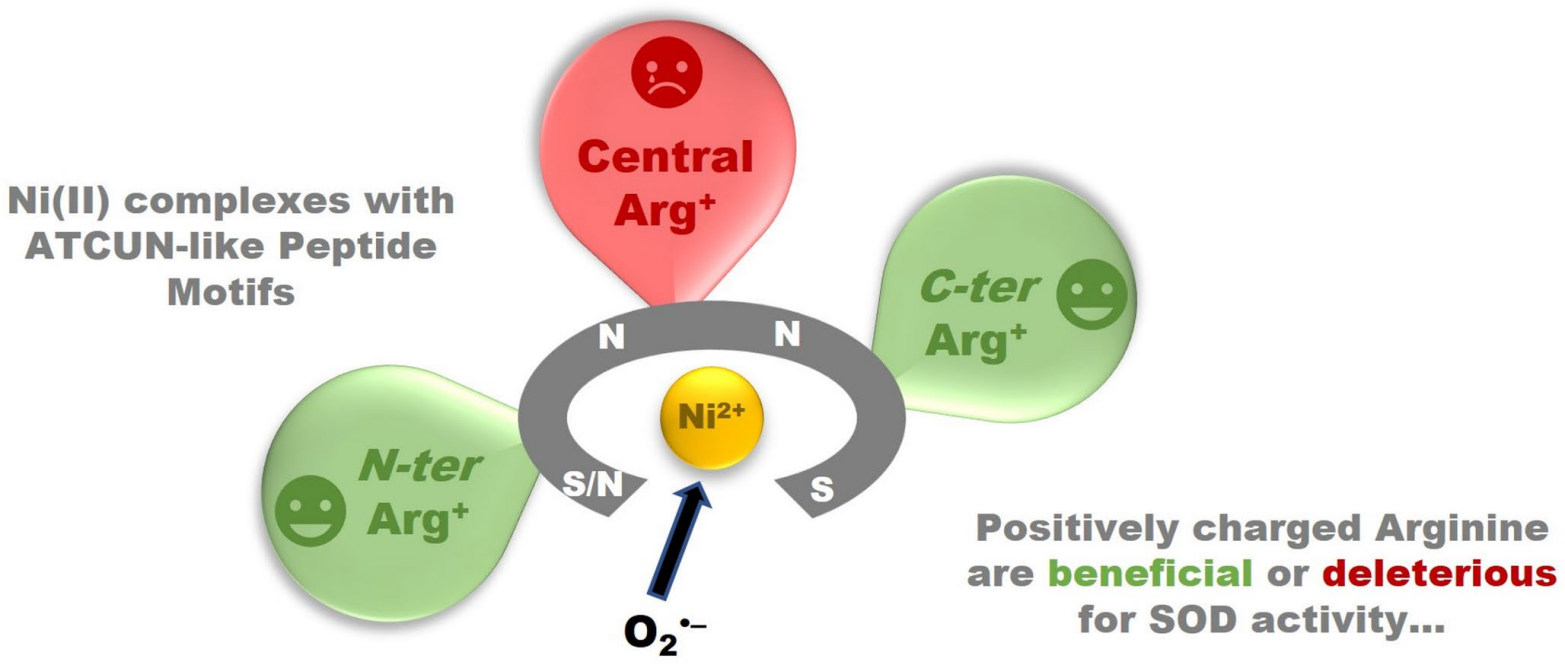

**Supplementary Information:**

The online version contains supplementary material available at 10.1007/s00775-026-02150-3.

## Introduction

Molecular dioxygen serves as the primary cellular electron acceptor during aerobic respiration, a process essential for energy production. However, this process also generates reactive oxygen species (ROS) as by-products through partial O_2_ reduction [1]. Among these ROS, the superoxide anion (O_2_°^−^) plays a dual role in cells: it acts as a signaling molecule in redox regulation and immune defense [2, 3], but when its levels are not tightly controlled, it contributes to oxidative stress, damaging biomolecules, and leading to cellular dysfunction. Oxidative stress is involved in aging, neurodegenerative disorders such as Parkinson’s and Alzheimer’s diseases, and various types of cancer [4].

**Fig. 1:**
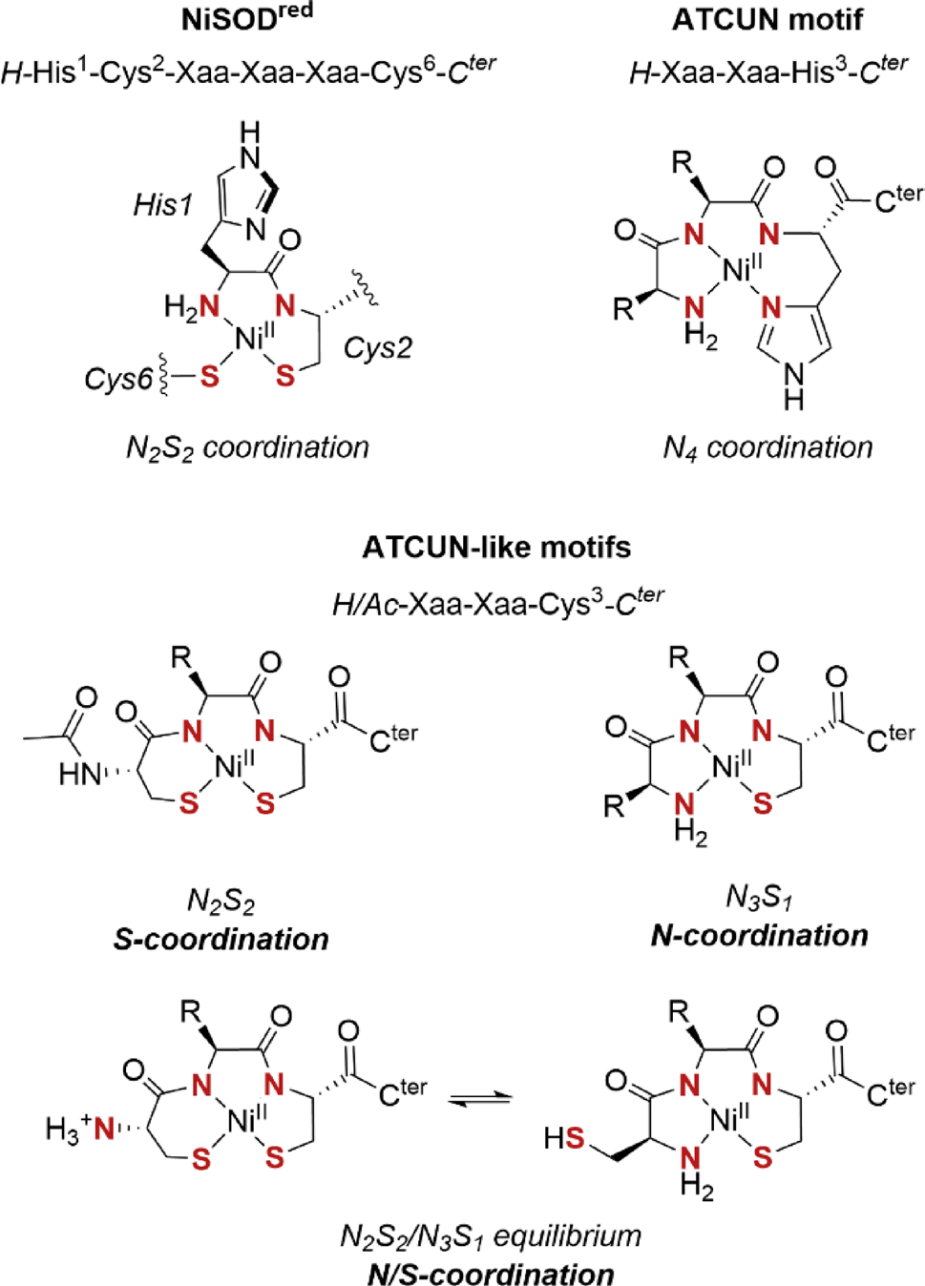
Coordination sites of the Ni(II) ion in the NiSOD, canonical ATCUN motif, and ATCUN-like motifs described in this work

To mitigate superoxide-induced damage, cells rely on superoxide dismutases (SODs), a family of metalloenzymes that catalyze the dismutation of superoxide into hydrogen peroxide (H_2_O_2_) and molecular dioxygen (O_2_). Different SODs are known and are generally classified based on the metal occupying their active site, namely Cu/Zn-SOD, Mn-SOD, and Fe-SOD. The Nickel-containing superoxide dismutase (NiSOD) is the most recently discovered variant, and is unique to certain bacteria and algae [5, 6]. NiSOD features a distinctive active site where the Ni(II) ion is coordinated by the terminal amine of histidine (His1), the amido nitrogen from the peptide bond (His1-Cys2), and two thiolates from cysteine residues (Cys2 and Cys6) (Fig. 1) [7]. This coordination results in a square-planar geometry for Ni(II), which evolves into a square-pyramidal geometry upon oxidation to Ni(III), as the imidazole group of His1 coordinates the metal ion as well [8]. The coordination sphere precisely tunes the Ni(III)/Ni(II) redox potential to lie between the reduction of superoxide to hydrogen peroxide and its oxidation to dioxygen, enabling both half-reactions.

Beyond its fundamental biochemical interest, the development of antioxidants remains a critical objective with bioinspired strategies representing a key avenue pursued by chemists [4]. In this context, elucidating the roles and relative importance of the various factors influencing the NiSOD activity is essential for unraveling its catalytic function and guiding the rational design of biomimetic complexes.

Efforts to develop NiSOD-inspired catalysts follow two main strategies: metallopeptides that mimic the NiSOD active site, and low-molecular-weight synthetic complexes designed to replicate its first coordination sphere [9–11]. Metallopeptides are water-soluble, and peptide ligand sequences can be readily tuned to explore structure-function relationships. However, peptides derived from the native NiSOD protein sequence exhibit several binding amino acids, which can lead to co-existing modes of coordination, and make the identification of the active species challenging [12, 13]. On the other hand, synthetic complexes have well-defined structures and coordination modes. However, most synthetic complexes to date have exhibited limited catalytic activity or have not been evaluated for SOD function [11, 14–16].

Notwithstanding, Ni(II) complexes derived from the Amino-Terminal Cu(II) and Ni(II) binding motif (ATCUN), a tripeptide sequence known to bind Cu(II) and Ni(II) at physiological pH, are a promising choice for mimicking the NiSOD active site (Fig. 1) [17–19]. Indeed, the ATCUN motif promotes a square-planar coordination, and the first coordination sphere of the metal can be easily tuned by altering the peptide sequence. For instance, replacing the histidine at the third position of the canonical ATCUN motif with a cysteine residue (ATCUN-like motifs, Fig. 1) was shown to generate a stable mononuclear Ni(II) complex, although its SOD activity was not assessed at the time [20].

**Fig. 2:**
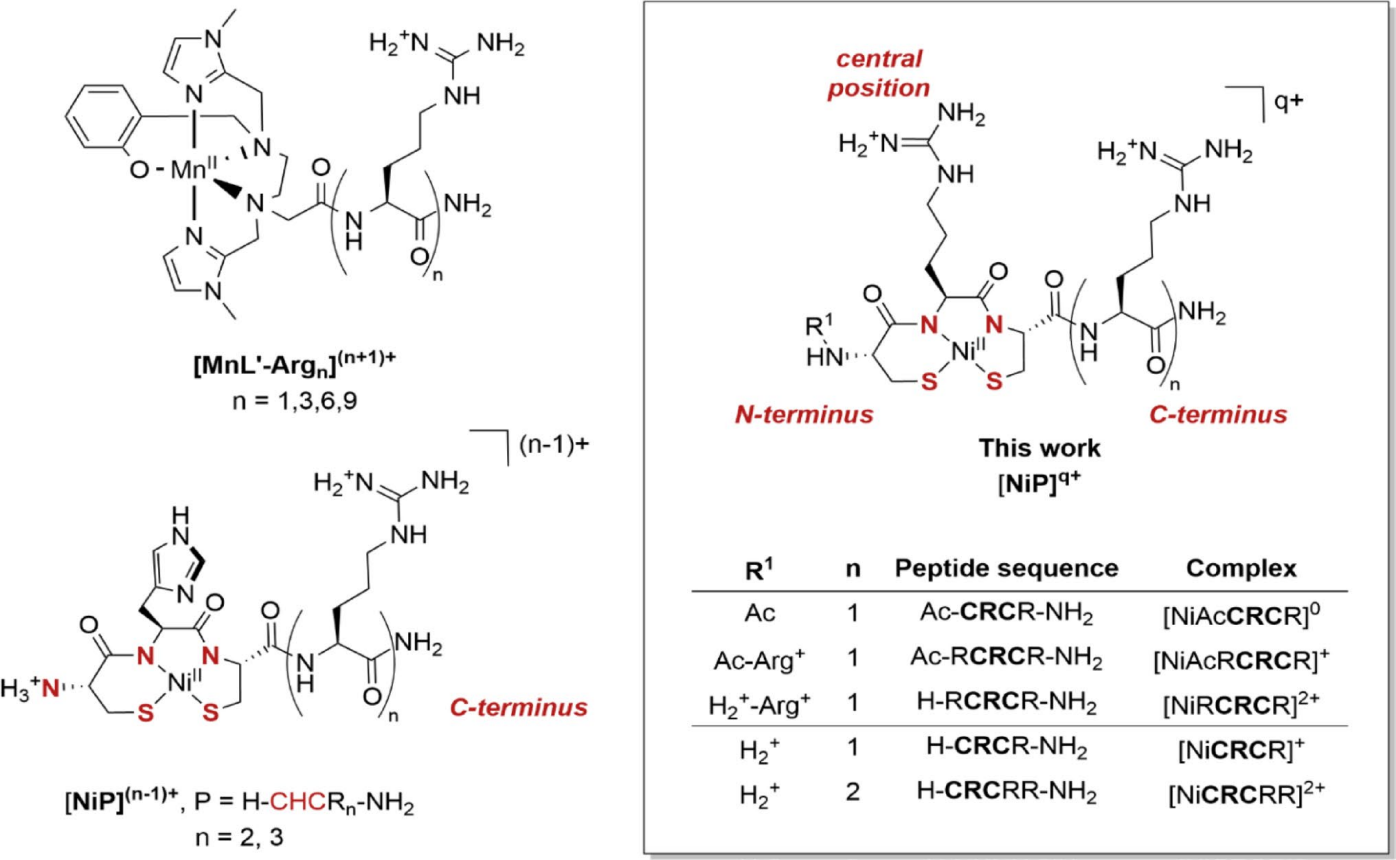
Examples of SOD mimics exhibiting diverse spatial positive charge distributions around the catalytic center [23, 24]. In the present work, positive charges were introduced at the central position of the ATCUN-like motif, as well as its C- and N-termini

We recently investigated ATCUN-like motifs tailored for SOD activity. Specifically, we explored the incorporation of one or two sulfur-containing residues, at the third position or the first and third positions, respectively. This led to Ni(II) complexes with different types of coordination spheres, i.e., N3S1 (*N*-coordination), N2S2 (*S*-coordination), and structures capable of interchanging between these two coordination modes with either the terminal amine (*N*-coordination) or thiolate (*S*-coordination) bound to Ni(II) (*N/S*-coordination, Fig. 1) [21, 22]. We identified key structural factors for optimal SOD activity [21–23]. Complexes endowed with the dynamic N/S-coordination mode exhibited the highest catalytic rates, likely due to proton transfer effects enhancing performance. Furthermore, incorporating a central histidine residue was also beneficial, as it stabilized the oxidized Ni(III) state as found in the enzyme (Fig. 2) [22, 23].

It has been shown that adding positive charges near the metal center, thereby mimicking the positively-charged substrate entrance channel of natural SODs, can enhance catalysis [23–29]. Among others, valuable work on MnSOD mimics has shown that functionalizing [MnL]^+^ with arginine residues near the metal center (Fig. 2) doubles catalytic rates with almost no alteration of redox properties [24]. Similarly, introducing positive charges at the C-terminus of ATCUN-like NiSOD mimics resulted in a remarkable enhancement of their SOD activity [23]. These previous works also showed that the distance of the charges to the metal center is key: positive charges close to the metal center did enhance SOD activity, while the addition of more distant arginine residues showed no further effect on catalysis [22–24]. However, in both cases, the positive charges were predominantly introduced unidirectionally, by elongating a poly-Arg sequence anchored to the SOD mimics at a specific position, at the periphery of the metal center. Positive charges were thus added at further distances from the catalytic center, but the impact of spatially arranging positive charges in close proximity to the metal center on its SOD activity was not investigated. To address this question, we took advantage of the versatility of the ATCUN-like motif, that enabled us to easily introduce positively charged residues not only at the C-terminus, but also at the N-terminus and central positions of the peptide (Fig. 2).

## Results And Discussion

### Design and synthesis of a series of ATCUN-like-based complexes

In order to investigate the effect of the spatial distribution of charges, we first introduced an arginine residue at the central position of the [CXC] binding motif (Fig. 2). This residue exhibits a guanidinium group that remains positively charged at physiological pH and does not coordinate metal ions. The positive charge was introduced via an Arg residue rather than a Lys residue, in order to avoid modification of the first coordination sphere through potential binding of the Lys primary amine to Ni(II) [30]. We also added one arginine residue at the C-terminus of the binding motif, since this modification proved beneficial on the SOD activity of similar complexes [23]. We then derivatized the N-terminus of the peptide with either an acetyl group, an acetylated arginine, or an arginine, thereby adding 0, 1 or 2 positive charges at the N-terminus, respectively. This led to the peptides Ac**CRC**R, AcR**CRC**R, and R**CRC**R, which are expected to form Ni(II) complexes with the so-called *S*-coordination, and a positive charge ranging from 0 to 2. Because the *N/S*-coordination was shown to be beneficial to SOD activity, we also included two peptides leading to this coordination mode in our study. For these peptides, the N-terminus could not be modified in order to preserve the equilibrium between the *S-* and *N*-coordination modes. We thus introduced one arginine residue at the central position and one or two arginine residues at the C-terminus. The resulting **CRC**R and **CRC**RR peptides are expected to yield Ni(II) complexes with a global positive charge of 1 and 2, respectively.

The ligands were synthesized using standard solid-phase peptide synthesis (SPPS) procedures (see Supporting Information and Figures S1-S10 for their purification and characterization). Since strong ligand-to-metal charge-transfer (LMCT) transitions from S^−^→Ni and N^−^→Ni were anticipated, the formation of the complexes was monitored by UV − vis spectroscopy, under an inert atmosphere to prevent thiol oxidation. Given that the ATCUN motif coordinates Ni(II) at physiological pH, peptide titrations were carried out in HEPES buffer at pH 7.4 (20 mM, [NaCl] = 100 mM).

All ligands lead to the rapid formation (within 5 min) of an initial species characterized by a low-energy band (λ_max_ ≈ 310 to 350 nm), indicating partial coordination of the ligand to the metal (Figures S11-S15) [31]. This initial species then evolves into a second species exhibiting a more intense charge transfer band at higher energy (λ_max_ ≈ 260 nm). For all five complexes, the transfer band displays a λ_max_ of about 260 nm and a ε_max_ in the 17–21 × 10^3^ L·mol^− 1^·cm^− 1^ range (Table 1), which is consistent with reported values for complexes with a similar first coordination sphere [22, 32]. A second band at 430–435 nm corresponding to a d − d transition evidences a square-planar geometry.

The formation of these ATCUN-like Ni(II) complexes occurs over the course of several hours. This is in line with the formation kinetics of previously reported similar Ni(II) complexes with a [CXC] ATCUN-like binding motif, which typically form within a few tens of minutes to a few hours [21–23]. This is yet slower than the related ATCUN complexes with a 4N coordination. Indeed, Cu(II)-ATCUN complexes typically form within 30 s, whereas their Ni(II) counterparts tend to form more slowly, within a few minutes to tens of minutes [33]. The nature of the amino acids in the peptide sequence can drastically impact the kinetics of the ATCUN-based complex formation [33, 34]. The presence of thiolate groups, as well as the nature of the other amino acids in the sequence, may thus contribute to the slow formation of the complexes.

Because of this, batch titrations were used to monitor the evolution of the spectroscopic signature for each sample over time, until stabilization.

**Table 1:** Propertiesof NiSOD mimics based on the ATCUN-like motif: UV-visible spectroscopic properties^a^ λ_max_ (nm) with the Molar Extinction Coefficients indicated in brackets, redox properties^b^ and reaction rate constants of the SOD-like activity^c^

Entry	Complex	Coordination	LMCT λ_max_ (nm),	d-d λ_max_ (nm),	*E*_p__a_ (V)	k_cat_^SOD^/10^5^ (M^− 1^.s^− 1^)	Ref
			(ε_max_/10^3^,M^− 1^.cm^− 1^)	(ε_max_/10^2^, M^− 1^.cm^− 1^)			
1	[NiAc**CAC**]^2−^	S	260 (24.3)	435 (2.0)	0.22	5.1(3)	[22]
2	[NiAc**CRC**]^−^	S	261 (21.0)	435 (2.0)	0.26	5.8(1)	[22]
3	[NiAc**CRC**R]^0^	S	262 (21.0)	435 (1.0)	0.26	9.2(5)	This work
4	[NiAcR**CRC**R]^+^	S	262 (18.0)	435 (1.0)	0.28	9.7(7)	This work
5	[NiR**CRC**R]^2+^	S	262 (17.0)	435 (1.5)	0.3	12.0(1)	This work
6	[Ni**CAC**]^−^	N/S	258 (19.9)	430 (3.0)	0.28	16(1)	[22]
7	[Ni**CRC**R]^+^	N/S	259 (20.0)	430 (0.8)	0.34	6.6(2)	This work
8	[Ni**CRC**RR]^2+^	N/S	259 (20.0)	430 (0.8)	0.41	6.6(1)	This work

^a^Values estimated in 20 mM HEPES buffer at pH 7.4, 100 mM NaCl.^b^ Estimated in 20 mM HEPES buffer at pH 7.4, 100 mM NaCl. Reference electrode: SCE.^c^Measured by stopped-flow experiments under catalytic conditions in 60 mM HEPES buffer, 100 mM NaCl, pH 8.1 and DMSO (5:1 v:v buffer/DMSO ratio).

## Effect of pH on the complexation of Ni(II)

**Fig. 3:**
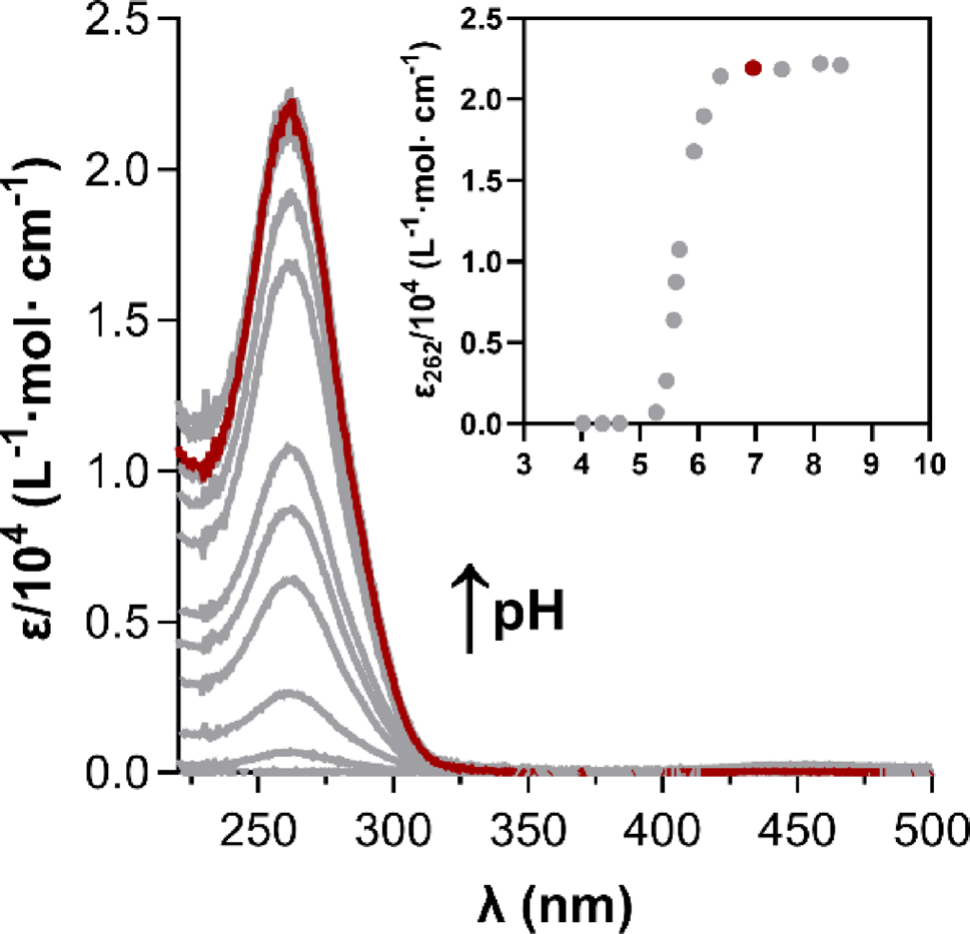
Effect of pH on the complexation of Ni(II) by AcRCRCR. [AcRCRCR] = 52 µM, [NiSO_4_] = 47 µM (0.9 eq.) in water, at different pH values. pH was adjusted with HCl or NaOH. Inset: Evolution of the absorbance of the LMCT band at 262 nm as a function of pH. The spectrum and ε_262_ at pH 7 are shown in red

The impact of pH on the Ni(II) complexation was investigated using UV − vis spectroscopy. This was achieved by adding 0.9 equivalents of Ni(II) to aqueous solutions of each ligand, with various concentrations of HCl or KOH. Spectra were recorded after the samples had stabilized over a period of 72 h for each pH. For all complexes, a plateau is reached above pH 6.5, confirming that all complexes are fully formed at physiological pH. Figure 3 illustrates a representative example of the ATCUN-like motifs used in this work, AcR**CRC**R, which will be used throughout the rest of the text. pH titrations and other characterizations for all other complexes can be found in the Supporting Information (Figures S16-S19).

## Formation of 1:1 Ni: ATCUN complexes

**Fig. 4:**
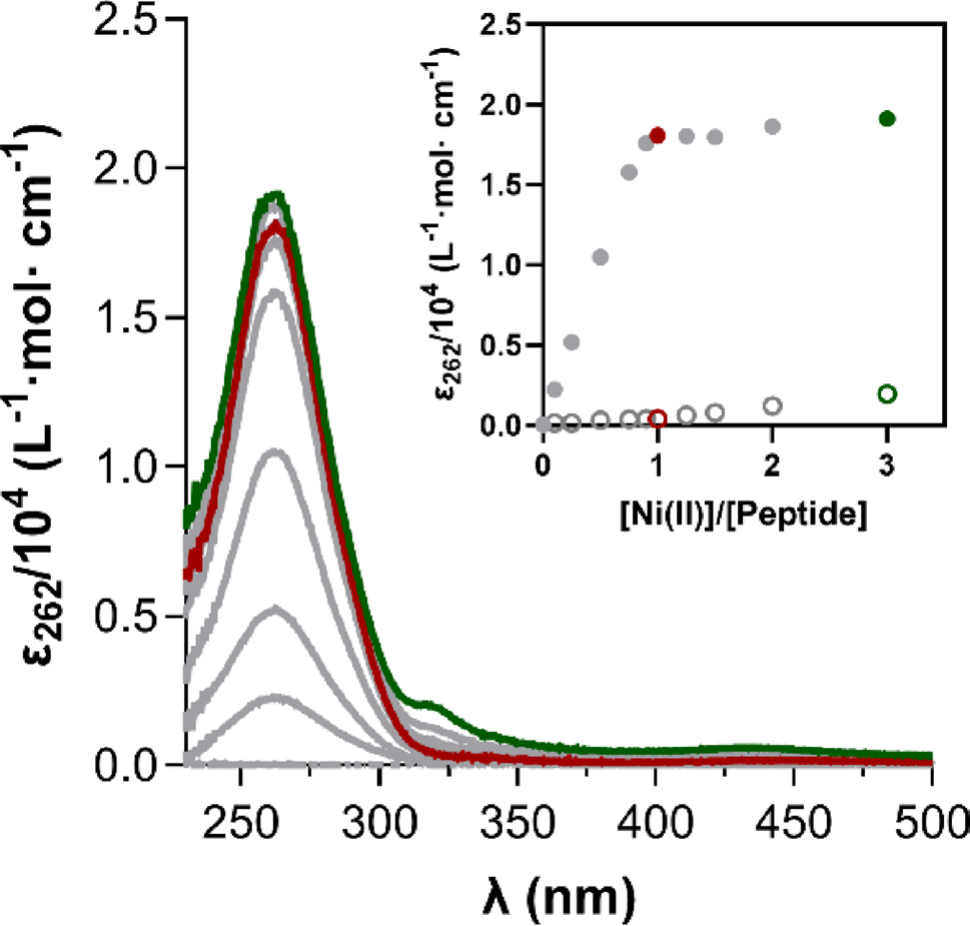
UV−vis spectra of [AcRCRCR] = 53 μM and increasing amounts of NiSO_4_ in HEPES buffer (20 mM, pH 7.4, [NaCl] = 100 mM). Inset: Absorbance evolution of the LMCT (full dots) and d-d band (empty circles) at 262 nm and 320 nm, respectively, as a function of the concentration of NiSO_4_. The spectrum, A_262_ and A_320_ values for the samples with 1 eq. Ni(II) and 3 eq. Ni(II) are shown in red and green, respectively

For each ligand, adding incremental amounts of NiSO_4_ in HEPES buffer (pH 7.4) results in a linear increase in absorbance at λ_max_ until 1 equivalent of Ni(II) is added (Fig. 4, Figures S20-S23), which is consistent with the exclusive formation of complexes with a 1:1 Ni: ligand ratio. When more than one equivalent of Ni(II) is added to *S*-ligands, a shoulder appears around 320 nm for [NiAc**CRC**R]^0^ and [NiAcR**CRC**R]^+^ (Fig. 4), and 335 nm for [NiR**CRC**R]^2+^, which aligns with previous studies on polymetallic complexes exhibiting similar coordination [35]. This behaviour, consistent with thiolates acting as bridging ligands between two metal ions, was also observed in previous studies on [NiAc**CAC**]^2−^ and [NiAc**CRC**]^−^ [22]. For *N/S*-complexes, a band of low intensity, likely due to d-d transitions on polymetallic species, appears around 400 nm beyond 1 equivalent of Ni(II), as observed for similar ATCUN-like Ni(II) complexes exhibiting this coordination [22, 23].

Electrospray ionization mass spectrometry (ESI-MS) studies were conducted in ammonium acetate (20 mM, pH 6.9), and confirmed the nature of the Ni(II) complexes identified through spectroscopic studies (Figures S24-S28). Only the mononuclear complex is detected when 0.9 equivalent of Ni is mixed with each peptide, but polymetallic species are formed when more than 1 equivalent of Ni is present in the mixture. For the peptides Ac**CRC**R and R**CRC**R, when more than 1 equivalent of Ni(II) is added, only polymetallic complexes, formed by two metal ions per ligand (2:1), are detected by ESI-MS.

Overall, these data indicate that after the addition of up to 1 equivalent of Ni(II) at physiological pH, all ligands yield a single mononuclear complex with a 1:1 stoichiometry. To prevent the formation of polymetallic complexes in subsequent experiments, a metal-to-ligand ratio of 0.9:1 was systematically employed.

## Superoxide dismutase activity of the complexes by stopped-flow

To evaluate the ability of the complexes to catalyze the dismutation of O_2_°^−^ into O_2_ and H_2_O_2_, the redox properties of each complex were investigated by cyclic voltammetry (CV) under an inert atmosphere. As reported for similar complexes, all complexes exhibit an irreversible oxidation process corresponding to the monoelectronic Ni(II) to Ni(III) oxidation (Figures S29 and S30) [21–23]. For *S*-complexes, the *E*_pa_ values for this Ni(III)/Ni(II) system are in the 0.26–0.3 V range vs. SCE (Table 1, entries 3–5). Consistent with the redox properties of [NiAc**CAC**]^−^ (*E*_pa_ = 0.22 V vs. SCE, Table 1, entry 6), a slight increase in the oxidation potential of the Ni center is observed as the global charge of the complexes increases. Overall, the redox properties of the Ni center fall within a suitable range for catalyzing the dismutation of superoxide, as they are lower than the reduction potential of superoxide while remaining higher than its oxidation potential (0.67 V and − 0.42 V vs. SCE, respectively) [4]. For *N/S*-complexes, both *S-* and *N*-coordination modes are in equilibrium in solution, the *S*-coordination mode being the main form. We previously demonstrated that complexes with a *S*-coordination display a decreased oxidation potential as compared to *N-*coordination, induced by an increased Ni(II) electronic density [22]. Consistently, *N/S*-complexes exhibit a similar trend to *S*-complexes, albeit with higher oxidation potentials, with *E*_pa_ values of 0.34 V vs. SCE for [Ni**CRC**R]⁺ and 0.41 V vs. SCE for [Ni**CRC**RR]^2^⁺. These values also fall within the appropriate potential range to achieve SOD activity (Table 1, entries 7 and 8). For this class of complexes, a second irreversible process is also observed at a more anodic potential, and attributed to the minor contribution of the complexes displaying the *N*-coordination mode [22].

The superoxide dismutase activity of the complexes was evaluated using stopped-flow experiments as previously reported [22]. Superoxide solution was prepared by mixing KO₂ in DMSO, with concentration determined by UV–vis spectroscopy. Measuring at pH 8.1 minimizes self-disproportionation, which follows a second-order rate law (k = 3(1) × 10^4^ M^− 1^ s^− 1^), consistent with reported data [36]. Upon addition of the Ni(II) complexes under catalytic conditions, the superoxide decay rate increases significantly, displaying a pseudo-first-order rate that is dependent of the complex concentration. The raw data from the stopped-flow experiments for all complexes and their analysis are presented in the Supporting Information (Figures S31-S35).

Given that superoxide and hydrogen peroxide can oxidize thiols, the complex stability was assessed by monitoring the LMCT band intensity after one second, corresponding to catalysis completion and superoxide full consumption. Minimal or no degradation was observed within this timeframe. When degradation was observed, the superoxide/complex ratio was adjusted accordingly. The resulting TON values are listed in Table S1.

**Fig. 5:**
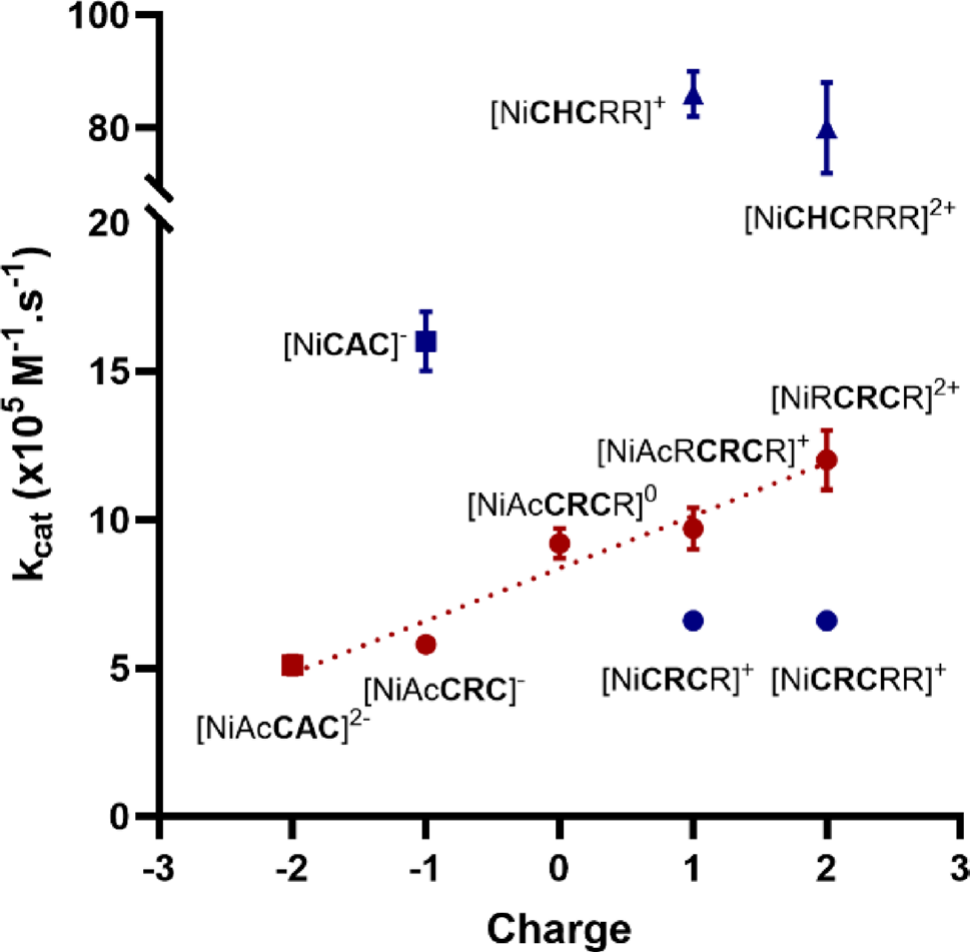
k_cat_ of the complexes studied in this work as a function of their charge, compared to the k_cat_ of the previously described complexes [NiAc**CAC**]^2-^, [NiAc**CRC**]^-^, [Ni**CAC**]^-^, [Ni**CHC**RR]^+^ and [Ni**CHC**RRR]^2+^.^22,23^ The standard deviations measured between the replicates of the measurements are indicated at each point. Values for S‑complexes are shown in red, for *N/S*‑complexes in blue. Complexes with the [CAC] motif are represented with squares, the [CRC] motif with circles and the [CHC] motif with triangles

Pseudo-first order *k*_cat_ values are reported in Table 1, entries 3–5 (*S*-complexes) and 7–8 (*N/S*-complexes). In the *S-*series, including the two previously reported complexes [NiAc**CAC**]^2−^ and [NiAc**CRC**]^−^ (entries 1 and 2), an increase in SOD activity is observed as the number of positive charges on the complexes increases (Fig. 5). However, this effect remains relatively modest, corresponding to an increase of the *k*_cat_ value by only a factor of 2.4 between [NiAc**CAC**]^2−^ and [NiR**CRC**R]^2+^. Surprisingly, both *N/S*-complexes [Ni**CRC**R]^+^ and [Ni**CRC**RR]^2+^exhibit unexpectedly low catalytic efficiencies (Table 1, entries 7–8), with *k*_cat_ values even lower than the one measured for the previously reported *N/S*-complex [Ni**CAC**]⁻, despite its global negative charge (Table 1, entry 6). Their SOD activity is ten-fold lower than that of the previously described [Ni**CHC**RR]^+^ and [Ni**CHC**RRR]^2+^, although they have the same charge and coordination mode (Fig. 5).

Altogether, these data show that introducing an arginine at the central position is highly detrimental for the SOD activity of the complexes, an effect that was only moderately balanced by introducing further positive charges at the C- and N-termini. The reasons for this drop in activity are unclear. One way to rationalize this observation is to invoke that the hindered Arg side chain prevents the superoxide anion to reach the catalytic site. This hypothesis is supported by the fact that upon Ni(II) binding, the ^1^H NMR signals of this central Arg, in particular its H^γ^ and H^δ^ protons, shift strongly downfield with very distinct chemical shifts for the two H^γ^ protons, features that were not found for C- and N-terminal Arg (Figure S36). This suggests some rigidification of the central Arg side chain, through electrostatic interactions or H-bond formation between the guanidinium and the metal center or its close environment.

There is also no clear correlation between redox potential and SOD activity of the NiSOD mimics. Similar to previously reported results [22], complexes with similar redox potential, charge, and coordination mode (e.g., [Ni**CHC**RR]^+^ and [Ni**CRC**R]^+^) display very different SOD activities. To perform the superoxide dismutation, the redox potential of the complexes must be in the range between the reduction of superoxide to hydrogen peroxide and its oxidation to dioxygen. Yet, while necessary, this condition is not sufficient to ensure an efficient SOD activity, highlighting further the multiplicity of factors involved. For instance, the performance of complexes exhibiting a His residue over others seems to indicate the importance of this residue for the catalysis. One can assume that, similarly to what is observed for the enzyme, the imidazole moiety binds to and consequently stabilizes the oxidized Ni(III) center during catalysis [7, 8, 37, 38].

## Design principles for ATCUN-based NiSOD mimics

Previous works on SOD mimics showed that introducing positive charges in the vicinity of the catalytic center was highly beneficial to SOD activity [23, 24]. In the present work, we exploited the versatility of the ATCUN peptide motif to introduce positively charged arginine residues in close proximity to the metal center, thereby investigating the impact of the spatial distribution of positive charges on SOD-like catalysis. Introduction of several Arg residues at the C-terminal position of the ATCUN-like motif has previously been shown to be highly beneficial, yielding a fourfold increase in catalytic efficiency [23]. Here, Arg residues were introduced at other positions, i.e., the central or N-terminal positions. Under stoichiometric conditions, all ATCUN-like ligands yield mononuclear Ni(II) complexes that are quantitatively formed at physiological pH. Complementary characterization techniques confirm the coordination of two amido and two thiolate groups to the nickel ion for the *S*-ligands, and two amido, one thiolate, and either another thiolate or one amine for the two *N/S-*ligands. As previously observed, such coordination spheres appropriately lower the oxidation potential of Ni(II), thereby mediating both the reduction and oxidation of superoxide and conferring catalytic SOD activity to these complexes. Their performance was evaluated under identical conditions to enable direct comparison of their *k*_cat_ values (Fig. 5).

By adjusting the global charge on a series of Ni complexes with *S*-coordination, we developed catalysts which can sustain multiple catalytic cycles with minimal degradation. This series demonstrates a clear beneficial effect of the charge increase from − 2 in [NiAc**CAC**]^2−^ to + 2 in [NiAcR**CRC**R]^2+^. However, this improvement appears to be limited by the critical positioning of the central arginine residue, as confirmed by the study of two other complexes that co-exist in *N*- and *S*-coordination modes. The results obtained with these two *N/S*-complexes evidence that the presence of an arginine residue in the central position of the ATCUN-like motif, not only fails to enhance activity but may actually impede catalysis. Indeed, the positively charged *N/S*-complexes, [Ni**CRC**R]^+^ and [Ni**CRC**RR]^2+^, were expected to show an SOD-like activity larger than their parent complex [Ni**CAC**]^−^, which bears a negative charge. Nevertheless, they demonstrated the lowest activity in the *N/S *series, indicating the deleterious impact of the central arginine on their SOD-like activity. An explanation arises from the bulky guanidinium group of arginine in the central position, which might lead to steric hindrance around the Ni center, obstructing its access to superoxide. Additionally, the local electrostatic environment induced by the Arg residues could influence protonation equilibria in ways that negatively impact the key proton-coupled electron-transfer steps required for efficient catalysis. Overall, the negative impact of the central arginine residue is most likely responsible for the very slight impact of charge on activity in this *S*-series. Although seemingly disappointing, these results remain informative for the future design of SOD mimics.

## Conclusions

These results emphasize the delicate balance required when modifying the ligand environment of Ni-based SOD mimics. While electrostatic interactions may play a significant role in catalysis, our findings highlight that the positioning of charged residues is of prime importance. The introduction of a central arginine in the ATCUN-like motif, initially intended to enhance substrate binding, instead appears to impede activity through a combination of steric hindrance and perturbation of proton transfer equilibria. This reinforces the idea that optimizing second-sphere interactions requires an intricate combination of parameters: approaching positive charges near the catalytic center is not sufficient if they are not spatially arranged properly. Indeed, bringing a positive charge too close to the catalytic center may even be detrimental to its activity.

However, this study also provides a clear path forward. Our previous work demonstrated that placing a histidine residue at the central ATCUN-like motif position dramatically boosts activity to ca. 10^7^ M^− 1^.s^− 1^, making it one of the most efficient synthetic NiSOD mimics reported to date [23]. On the contrary, we demonstrate here that an Arg at the same central position is deleterious to activity. Along with results establishing that the *N/S*-coordination mode enhances activity, these findings define a clear set of design principles: the N-terminal extremity must remain free to maintain this coordination mode, the central residue must be a histidine, and while the C-terminal region can carry a charge, only residues in direct proximity to the metal center significantly influence catalysis. This work not only refines our understanding of NiSOD-inspired catalysis but also provides a robust foundation for the rational design of future biomimetic catalysts.

## Methods

Procedures for peptide synthesis, analytical and preparative HPLC, UV-visible spectroscopy, mass spectrometry, ^1^H NMR spectroscopy, electrochemistry and stopped flow experiments were identical to the ones previously described for similar ATCUN-like NiSOD mimics [22, 23].

## UV-visible spectroscopy titrations

Due to the slow complex formation, pH studies and Ni titrations were performed in batch: for each point reported in the Main text and the Supporting Information, a solution with the appropriate amount of peptide, nickel, and base, acid, or buffer was prepared in a distinct cuvette. All solutions were left to equilibrate for several hours, and their absorbance and pH (for pH titrations) were measured until equilibrium was reached, i.e., no evolution of the absorbance or the pH (for pH titrations) was detected.

### Stopped-flow data analysis

Stopped-flow experiments were performed as previously reported [22, 23]. For each complex concentration (2.5 to 50 µM), the UV traces of 5–9 shots were averaged, and k_obs_ was obtained by fitting this average trace following a pseudo-first order reaction model [O_2_°^−^] (t) = [O_2_°^−^]_0_ e^−kobs t^. The k_obs_ values were used to determine k_cat_, using the following equation: k_obs_ = k_cat_ [complex]. The whole experiment was performed twice, and the mean and standard deviation of the resulting k_cat_ values are reported here.

## Electronic Supplementary Material

Below is the link to the electronic supplementary material.

Supplementary Material 1

## Data Availability

All data supporting the findings of this study are available within the paper and its Supplementary Information.
