# Supplementary material for: Impact of the spatial distribution of positive charges on the catalytic activity of NiSOD bioinspired complexes
Source: J Biol Inorg Chem. 2026 May 19;31(4):303–12. doi: 10.1007/s00775-026-02150-3 (PMC13356053; doi:10.1007/s00775-026-02150-3)
Supplement: Supplementary file 1 — Supplementary Material 1 [file 775_2026_2150_MOESM1_ESM.pdf]

Supporting Information

Impact of the Spatial Distribution of Positive Charges on the Catalytic Activity of NiSOD Bioinspired Complexes

Pawel Guinard,[a,b] Jacques Pécaut,[a] Alan Le-Goff,[b] Carole Duboc,*[b] Pascale Delangle,*[a] Sarah Hostachy*[a]

[a] Dr P. Guinard, Dr J. Pécaut, Dr P. Delangle*, Dr S. Hostachy*

Univ. Grenoble Alpes, CEA, CNRS, Grenoble INP, IRIG, SyMMES, 38000 Grenoble, France

sarah.hostachy@cea.fr, pascale.delangle@cea.fr

[b] Dr P. Guinard, Dr A. Le-Goff, Dr C. Duboc*

Univ. Grenoble Alpes, CNRS, DCM, 38000 Grenoble, France

carole.duboc@univ-grenoble-alpes.fr

Table of contents

1. Peptide synthesis and characterization 3
   1.1. AcCRCR 4
   1.2. AcRCRCR 5
   1.3. RCRCR 6
   1.4. CRCR 7
   1.5. CRCRR 8
2. Kinetics of complex formation 9
3. pH dependence of complex formation 11
4. Ligand titration with Ni(II) 13
   4.1. UV-visible spectroscopy: 13
   4.2. ESI-MS 15
5. Electrochemistry 17
6. Catalytic activity of the complexes 18
7. ^{1}H NMR characterization of $[NiCRCR]^{+}$ 21

1. Peptide synthesis and characterization

Purified ligands were analyzed using a Chromolith® Performance RP-18 column (100-4.6 mm, with macropore size of 200 Å and mesopore size of 13 Å from Merk Millipore), equipped with a Chromolith® guard column.

HPLC solvents contained either trifluoroacetic acid (TFA) or formic acid (FA):

- **A:** H_2O/TFA (99.9/0.1), **B:** ACN/H_2O/TFA (90/9.9/0.1);
- **C:** H_2O/FA (99.9/0.1), **D:** ACN/H_2O/FA (90/9.9/0.1).

Gradient 1: 0% B for 7 min, 0 to 100% B in 5 min, 100% for 5 min.

Gradient 2: 5% B for 5 min, 5 to 100% B in 10 min, and 100% B for 3 min.

Gradient 3: 5% C for 5 min, 5 to 100% D in 5 min, and 100% D for 5 min.

1.1. AcCRCR

Yield : 14%

HPLC: gradient 2, t_R = 3.5 min, 97% purity.

ESI-MS (positive mode): M = $C_{20}H_{39}N_{11}O_5S_2$, m/z calculated for $[M+H]^+$ 578.3, found 578.3.

^{1}H NMR: (H_2O/D_2O 90:10, 500 MHz, 298 K, pH = 7.6) δ ppm = 1.58 (m, 4H, $H_{\gamma,Arg}$), 1.70 (m, 2H, $H_{\beta,Arg}$), 1.82 (m, 2H, $H_{\beta,Arg}$), 1.96 (s, 3H, $CH_{3\ Nter}$), 2.81 (m, 2H, $H_{\beta,Cys}$), 2.85 (m, 2H, $H_{\beta,Cys}$), 3.13 (m, 4H, $H_{\delta,Arg}$), 4.21 (m, 1H, $H_{\alpha,Arg}$), 4.29 (m, 1H, $H_{\alpha,Arg}$), 4.31 (m, 1H, $H_{\alpha,Cys}$), 4.35 (m, 1H, $H_{\alpha,Cys}$), 7.08 (bs, 1H, $NH_{2\ Cter}$), 7.57 (bs, 1H, $NH_{2\ Cter}$).

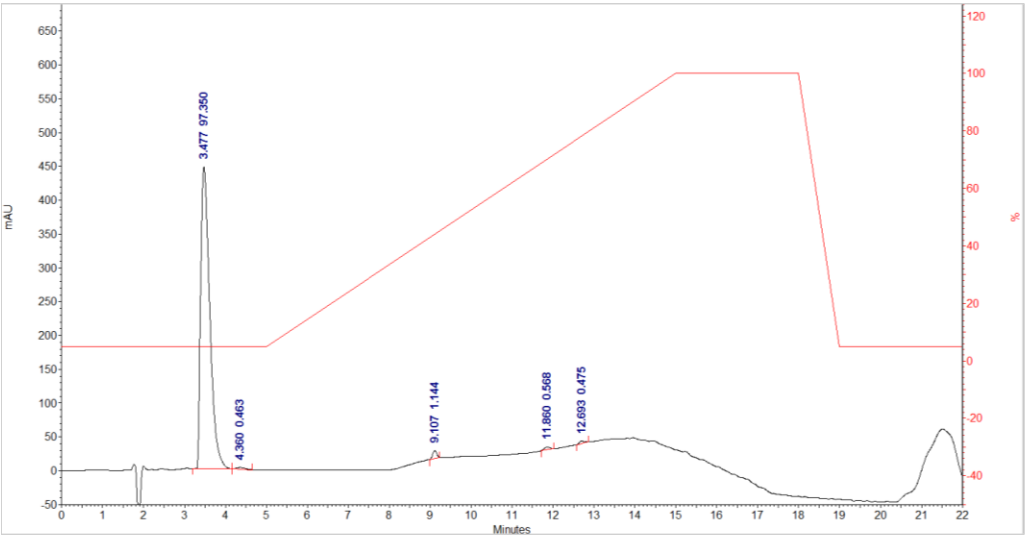

Figure S1. Analytical HPLC chromatogram of purified AcCRCR, gradient 2 (see Main Text).

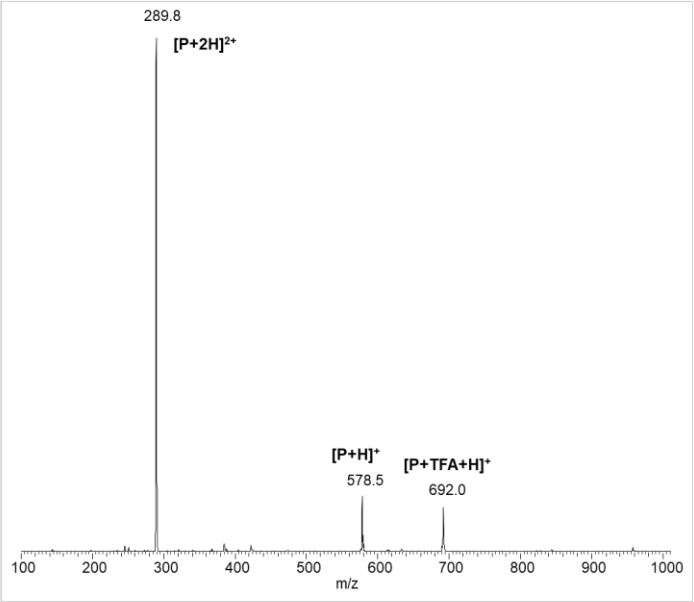

Figure S2.ESI-MS (+) spectrum of AcCRCR

1.2. AcRCRCR

Yield : 30%

HPLC: gradient 1, t_R = 2.7 min, 93% purity.

ESI-MS (positive mode): M = $C_{26}H_{51}N_{15}O_6S_2$, m/z calculated for $[M+H]^+$ 733.4, found 733.7.

^{1}H NMR: (H_2O/D_2O 90:10, 500 MHz, 298 K, pH = 7.1) δ ppm = 1.57 (m, 6H, $H_{\gamma,Arg}$), 1.70 (m, 4H, $H_{\beta,Arg}$), 1.80 (m, 2H, $H_{\beta,Arg}$), 1.95 (s, 3H, $CH_{3\ Nter}$), 2.85 (m, 4H, $H_{\beta,Cys}$), 3.12 (m, 6H, $H_{\delta,Arg}$), 4.20 (m, 2H, $H_{\alpha,Arg}$), 4.26 (m, 1H, $H_{\alpha,Arg}$), 4.36 (m, 2H, $H_{\alpha,Cys}$), 7.08 (bs, 1H, $NH_{2\ Cter}$), 7.57 (bs, 1H, $NH_{2\ Cter}$).

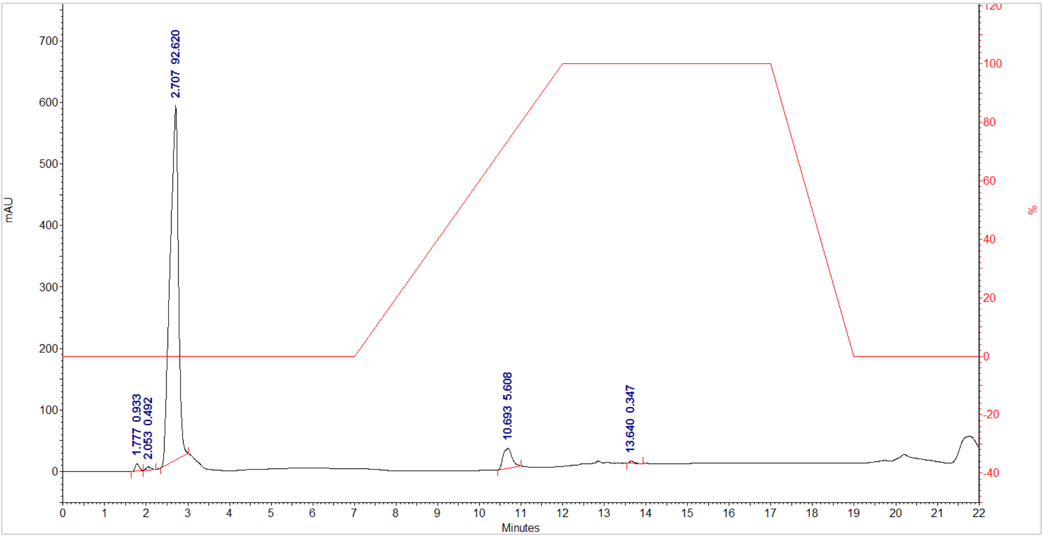

Figure S3. Analytical HPLC chromatogram of purified AcRCRCR, gradient 1 (see Main Text).

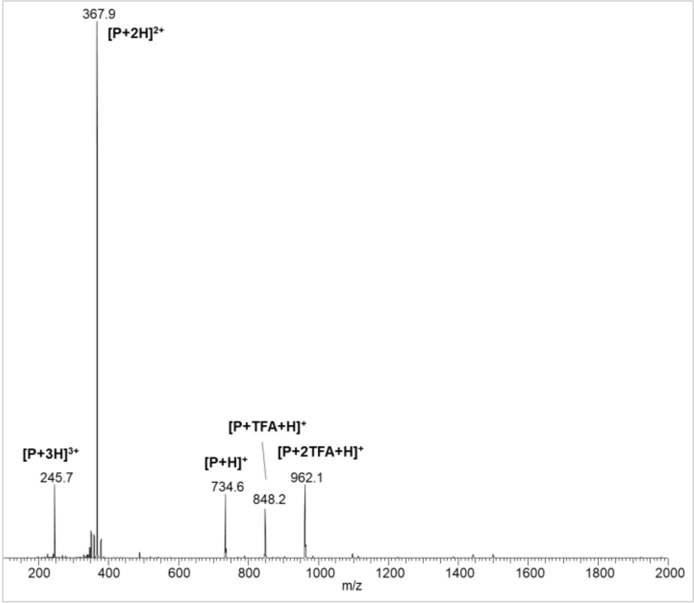

Figure S4. ESI-MS (+) spectrum of AcRCRCR. P stands for the neutral peptide.

1.3. RCRCR

RCRCR, yield : 14%

HPLC: gradient 1, t_R = 2.1 min, 97% purity.

ESI-MS (positive mode): M = $C_{24}H_{49}N_{15}O_5S_2$, m/z calculated for $[M+H]^+$ 692.4, found 692.4.

^{1}H NMR: (H_2O/D_2O 90:10, 500 MHz, 298 K, pH = 7.3) δ ppm = 1.57 (m, 6H, $H_{\gamma,Arg}$), 1.70 (m, 2H, $H_{\beta,Arg}$), 1.80 (m, 4H, $H_{\beta,Arg}$), 2.86 (m, 4H, $H_{\beta,Cys}$), 3.13 (m, 6H, $H_{\delta,Arg}$), 3.96 (m, 1H, $H_{\alpha,Arg}$), 4.19 (m, 1H, $H_{\alpha,Arg}$), 4.26 (m, 1H, $H_{\alpha,Arg}$), 4.42 (m, 1H, $H_{\alpha,Cys}$), 4.46 (m, 1H, $H_{\alpha,Cys}$), 7.08 (bs, 1H, $NH_{2\ Cter}$), 7.55 (bs, 1H, $NH_{2\ Cter}$).

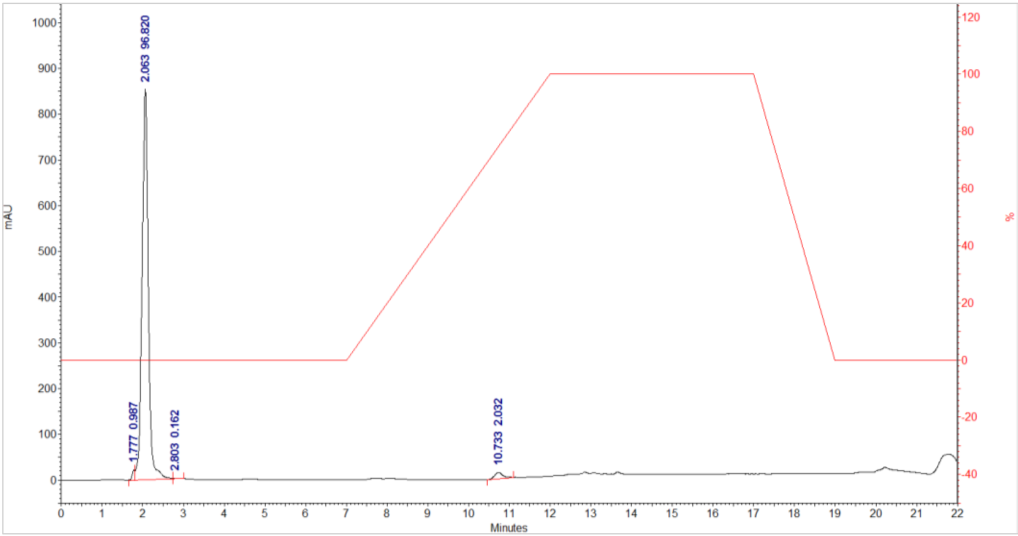

Figure S5. Analytical HPLC chromatogram of purified RCRCR, gradient 1 (see Main Text).

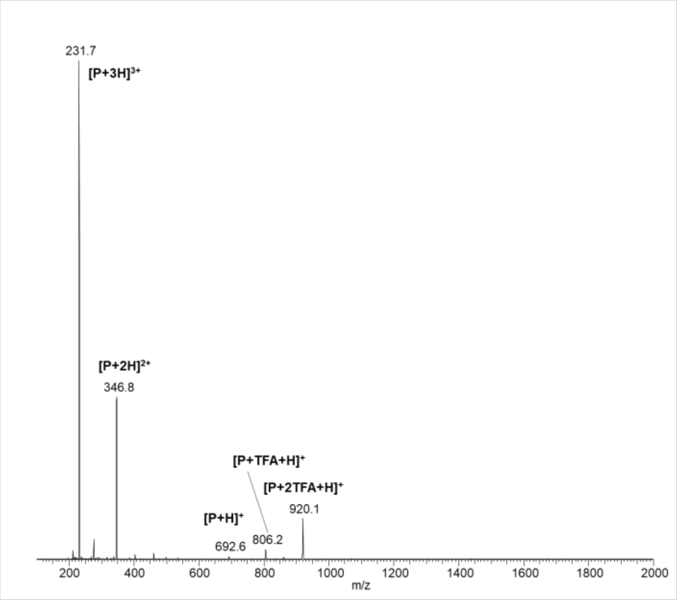

Figure S6. ESI-MS (+) spectrum of RCRCR.

1.4. CRCR

CRCR, yield : 13%

HPLC: gradient 3, t_R = 1.6 min, 99% purity.

ESI-MS (positive mode): M = $C_{18}H_{37}N_{11}O_4S_2$, m/z calculated for $[M+H]^+$ 536.3, found 536.4.

^{1}H NMR: (H_2O/D_2O 90:10, 500 MHz, 298 K, pH = 7.1) δ ppm = 1.57 (m, 4H, $H_{\gamma,Arg}$), 1.71 (m, 2H, $H_{\beta,Arg}$), 1.78 (m, 2H, $H_{\beta,Arg}$), 2.86 (d, *J* = 6.5 Hz, 2H, $H_{\beta,Cys}$), 2.95 (ABX, J_{AB} = 14.7 Hz, J_{AX} = 5.5 Hz, 1H, $H_{\beta,Cys}$), 3.01 (ABX, J_{AB} = 14.7 Hz, J_{BX} = 5.6 Hz, 1H, $H_{\beta,Cys}$), 3.13 (m, 4H, $H_{\delta,Arg}$), 4.12 (bt, *J* = 5.6 Hz, 1H, $H_{\alpha,Cys}$), 4.20 (m, 1H, $H_{\alpha,Arg}$), 4.31 (m, 1H, $H_{\alpha,Arg}$), 4.41 (m, 1H, $H_{\alpha,Cys}$), 7.08 (s, 1H, $NH_{2\ Cter}$), 7.54 (s, 1H, $NH_{2\ Cter}$).

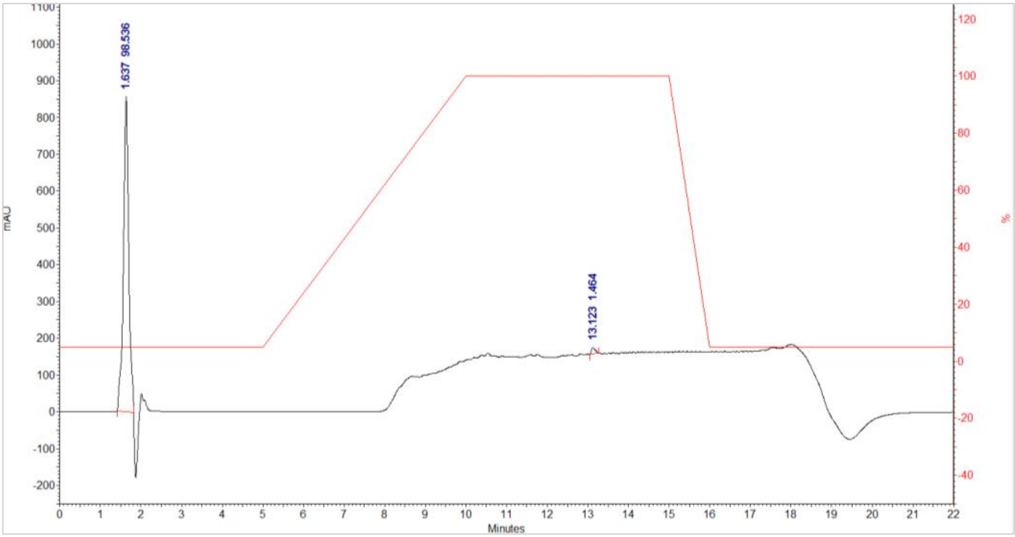

Figure S7. Analytical HPLC chromatogram of purified CRCR, gradient 3 (see Main Text).

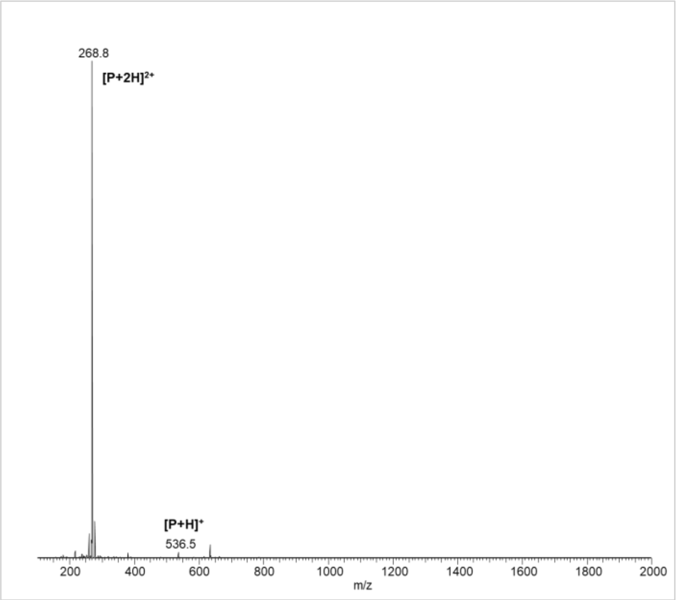

Figure S8. ESI-MS (+) spectrum of CRCR.

1.5. CRCRR

Yield : 15%

HPLC: gradient 3, t_R = 1.6 min, 95% purity.

ESI-MS (positive mode): M = $C_{24}H_{49}N_{15}O_5S_2$, m/z calculated for $[M+H]^+$ 692.4, found 692.5.

^{1}H NMR: (H_2O/D_2O 90:10, 500 MHz, 298 K, pH = 5.5) δ ppm = 1.57 (m, 6H, $H_{\gamma,Arg}$), 1.70 (m, 3H, $H_{\beta,Arg}$), 1.78 (m, 3H, $H_{\beta,Arg}$), 2.84 (d, *J* = 6.60 Hz, 2H, $H_{\beta,Cys}$), 2.89 (m, 2H, $H_{\beta,Cys}$), 3.12 (m, 6H, $H_{\delta,Arg}$), 3.92 (m, 1H, $H_{\alpha,Cys}$), 4.21 (m, 1H, $H_{\alpha,Arg}$), 4.26(m, 1H, $H_{\alpha,Arg}$), 4.29 (m, 1H, $H_{\alpha,Arg}$), 4.39 (m, 1H, $H_{\alpha,Cys}$), 7.04 (s, 1H, $NH_{2\ Cter}$), 7.57 (s, 1H, $NH_{2\ Cter}$).

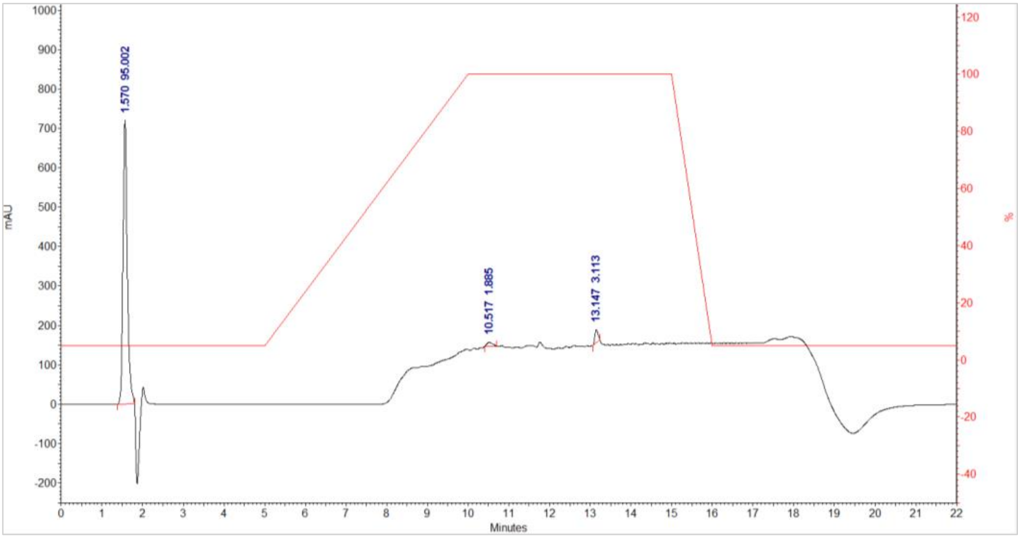

Figure S9. Analytical HPLC chromatogram of purified CRCRR, gradient 3 (see Main Text).

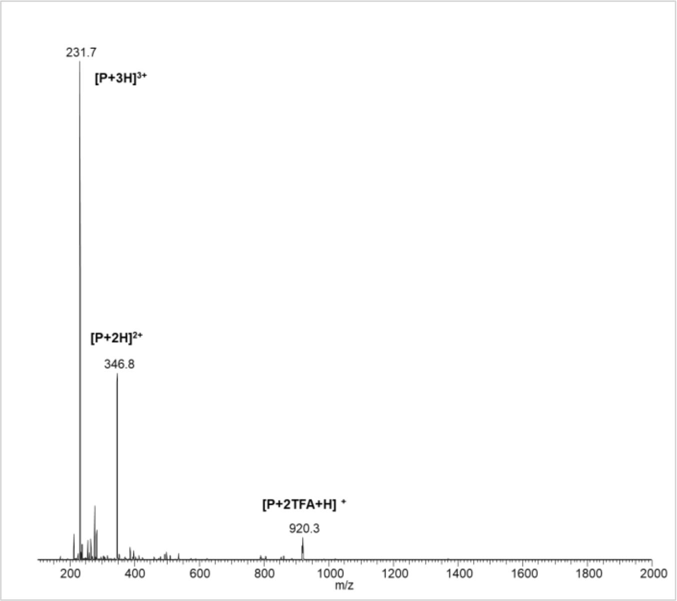

Figure S10. ESI-MS (+) spectrum of CRCRR.

2. Kinetics of complex formation

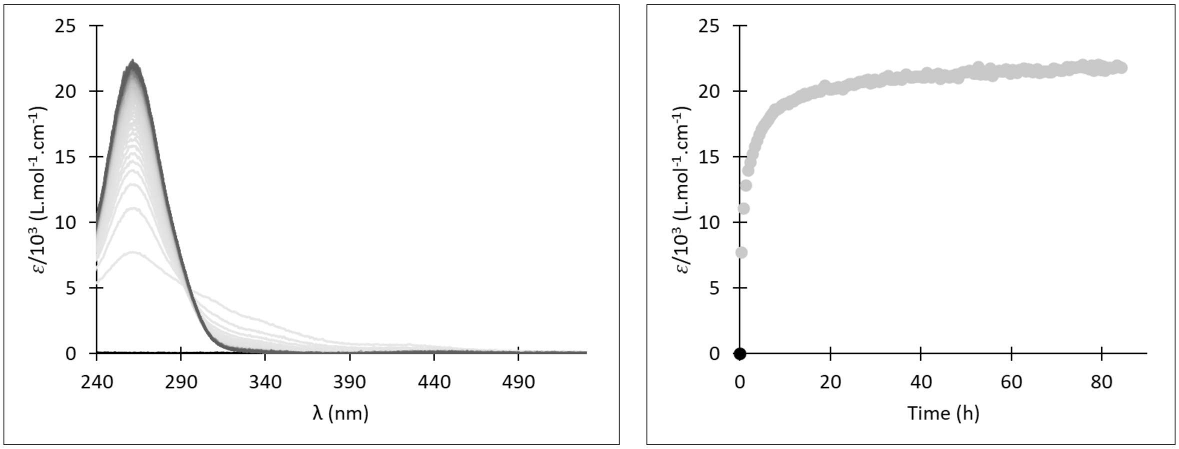

Figure S11. Kinetics of [NiAcCRCR]^{0} formation. *Left: UV-visible spectra at different times of a solution of AcCRCR (56 µM) with 0.9 équiv.* $NiSO_4$ *in HEPES buffer (20 mM, pH 7.4, [NaCl] = 0.1 M). Right: Absorbance evolution at 262 nm with time (h).*

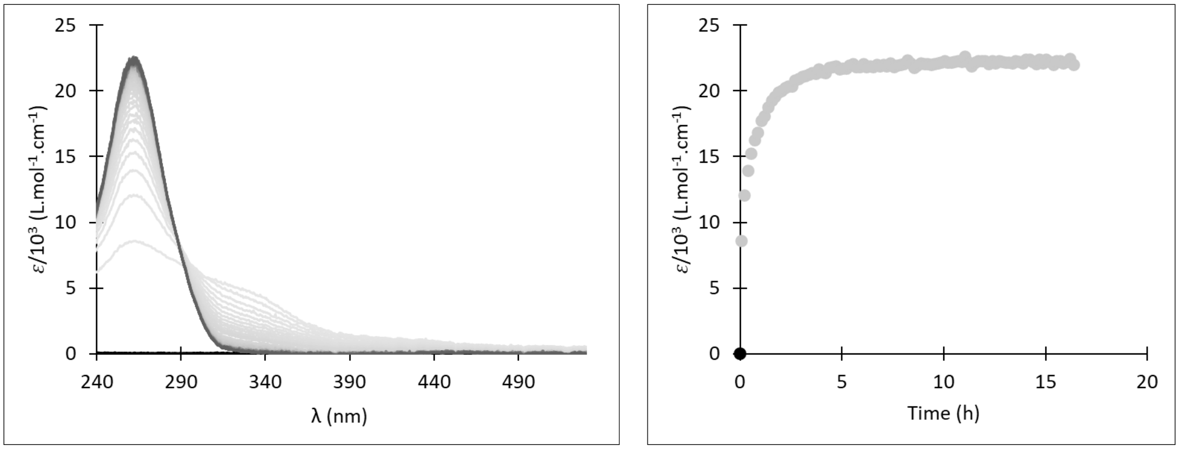

Figure S12. Kinetics of [NiAcRCRCR]$^{+}$ formation. *Left: UV-visible spectra at different times of a solution of AcRCRCR (56 µM) with 0.9 équiv.* $NiSO_4$ *in HEPES buffer (20 mM, pH 7.4, [NaCl] = 0.1 M). Right: Absorbance evolution at 262 nm with time (h).*

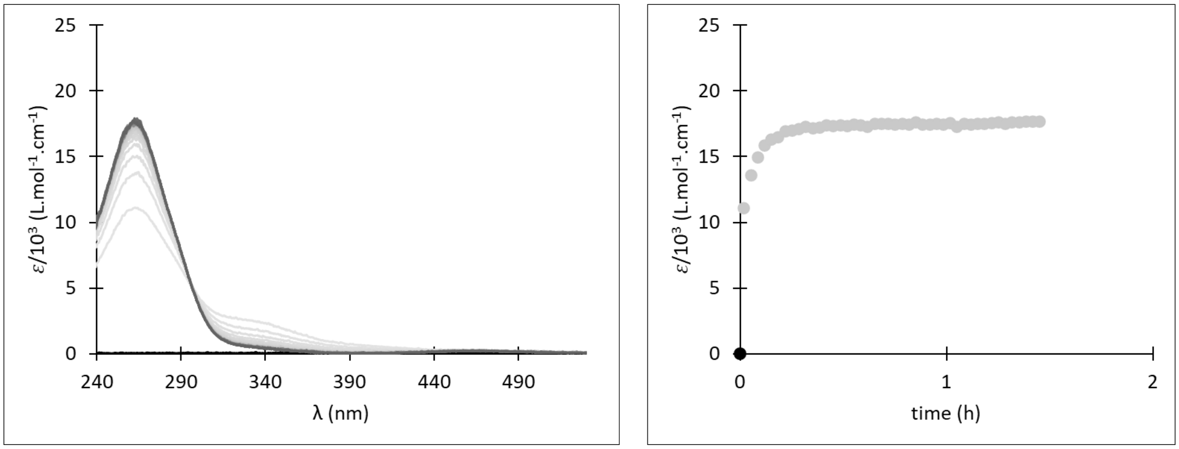

Figure S13. Kinetics of [NiRCRCR]$^{2+}$ formation. *Left: UV-visible spectra at different times of a solution of RCRCR (56 µM) with 0.9 équiv. $NiSO_4$ in HEPES buffer (20 mM, pH 7.4, [NaCl] = 0.1 M). Right: Absorbance evolution at 262 nm with time (h).*

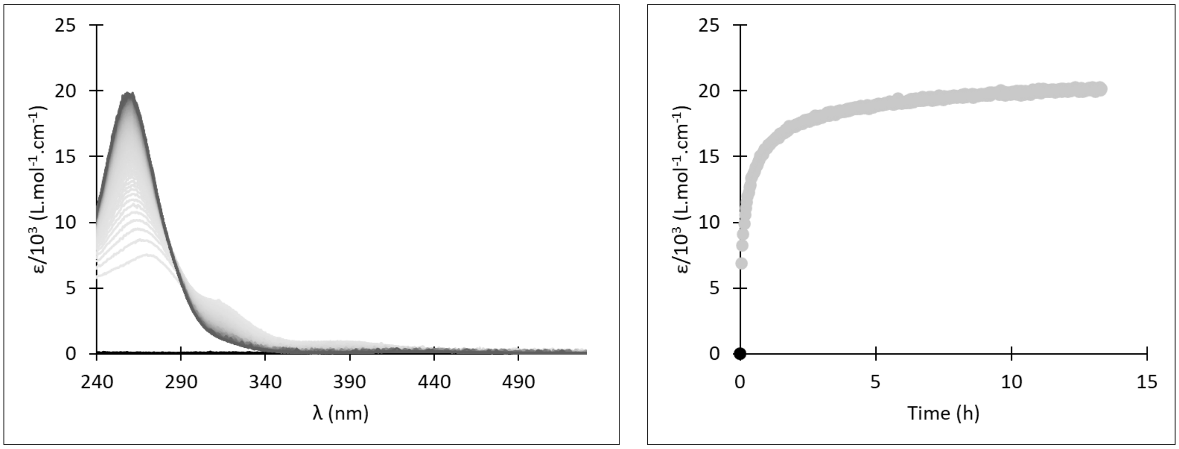

Figure S14. Kinetics of [NiCRCR]$^{+}$ formation. *Left: UV-visible spectra at different times of a solution of CRCR (56 µM) with 0.9 équiv. $NiSO_4$ in HEPES buffer (20 mM, pH 7.4, [NaCl] = 0.1 M). Right: Absorbance evolution at 259 nm with time (h).*

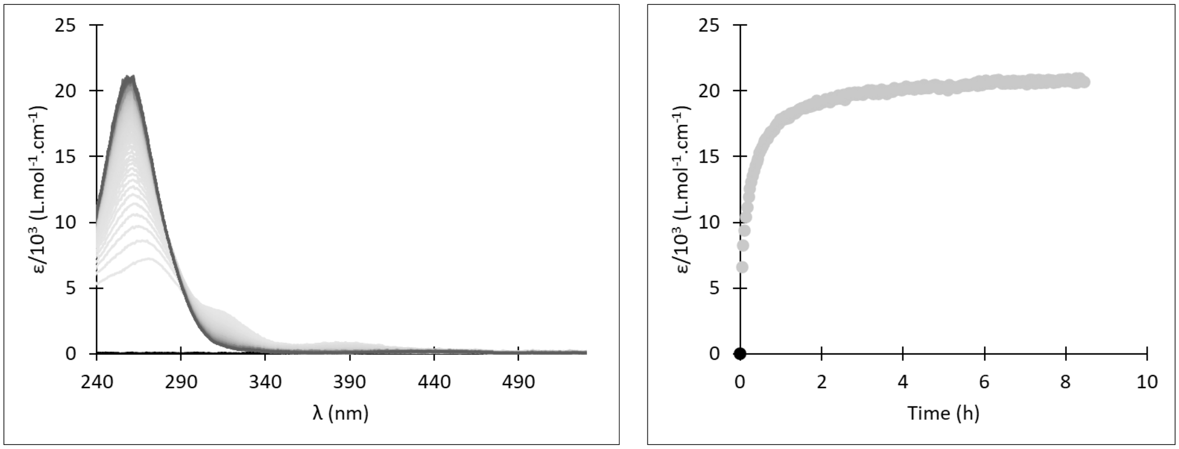

Figure S15. Kinetics of [NiCRCRR]$^{2+}$ formation. *Left: UV-visible spectra at different times of a solution of CRCRR (56 µM) with 0.9 équiv. $NiSO_4$ in HEPES buffer (20 mM, pH 7.4, [NaCl] = 0.1 M). Right: Absorbance evolution at 259 nm with time (h).*

3. pH dependence of complex formation

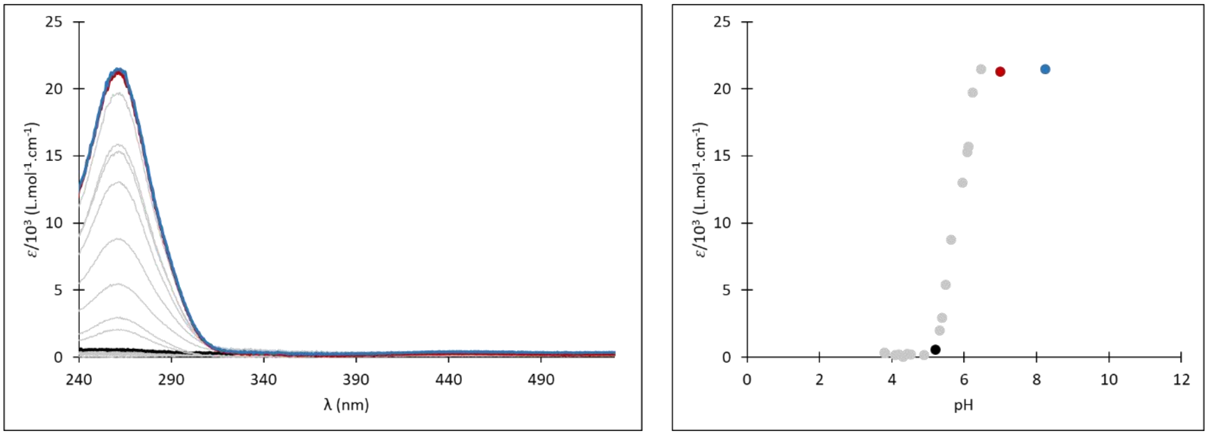

***Figure S16. pH dependance of [NiAcCRCR]^{0} formation**. Left: UV-vis spectra of AcCRCR (45 µM) with 0.9 equiv. $NiSO_4$ in water as a function of pH. Right: absorbance evolution at 262 nm as a function of pH. Spectra and A_{262} at pH 5.2, 7 and 8.2 are shown in black, red and blue, respectively.*

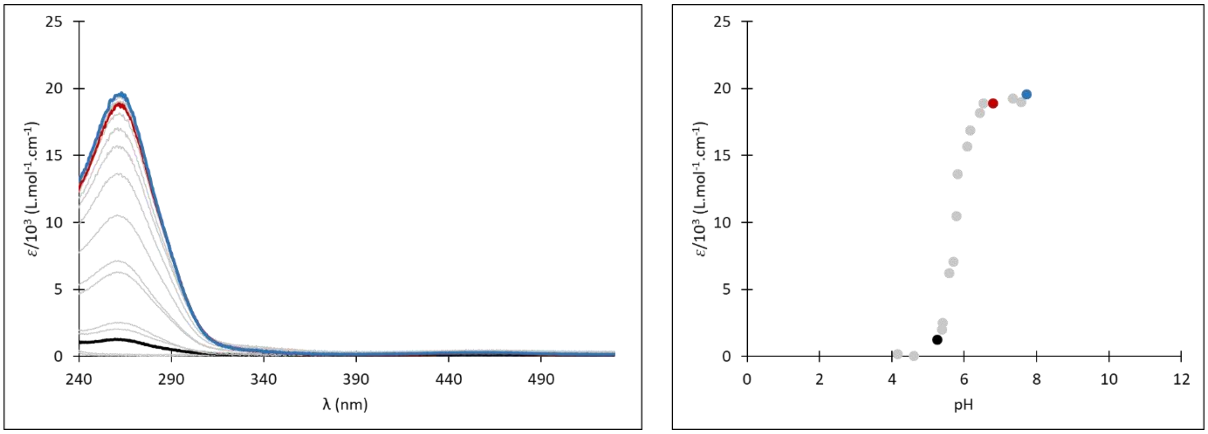

***Figure S17. pH dependance of [NiRCRCR]$^{+}$ formation.** Left: UV-vis spectra of RCRCR (53 µM) with 0.9 equiv. $NiSO_4$ in water as a function of pH. Right: absorbance evolution at 262 nm as a function of pH. Spectra and A_{262} at pH 5.3, 6.8 and 7.7 are shown in black, red and blue, respectively.*

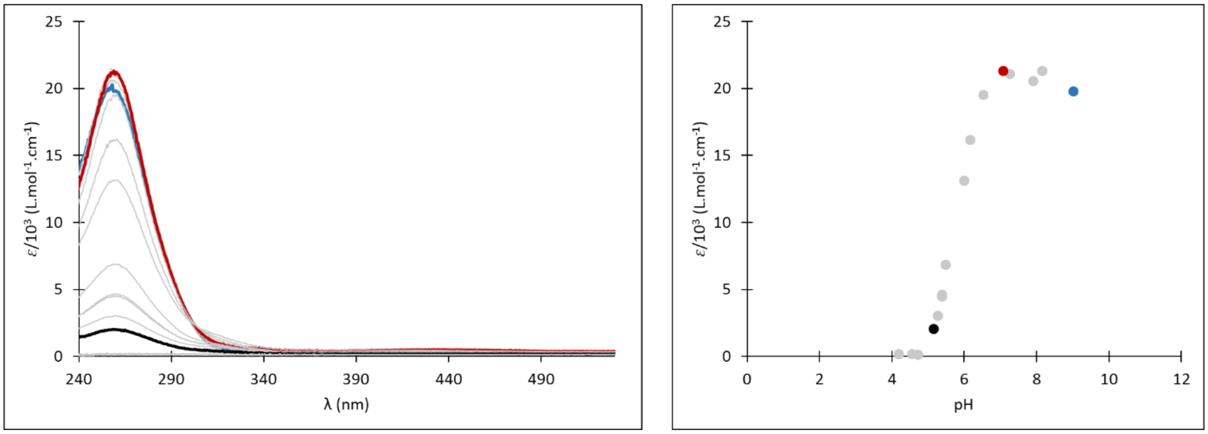

Figure S18. pH dependance of [NiCRCR]$^{+}$ formation. *Left: UV-vis spectra of CRCR (50 µM) with 0.9 equiv. $NiSO_4$ in water as a function of pH. Right: absorbance evolution at 259 nm as a function of pH. Spectra and A_{262} at pH 5.2, 7.1 and 9 are shown in black, red and blue, respectively.*

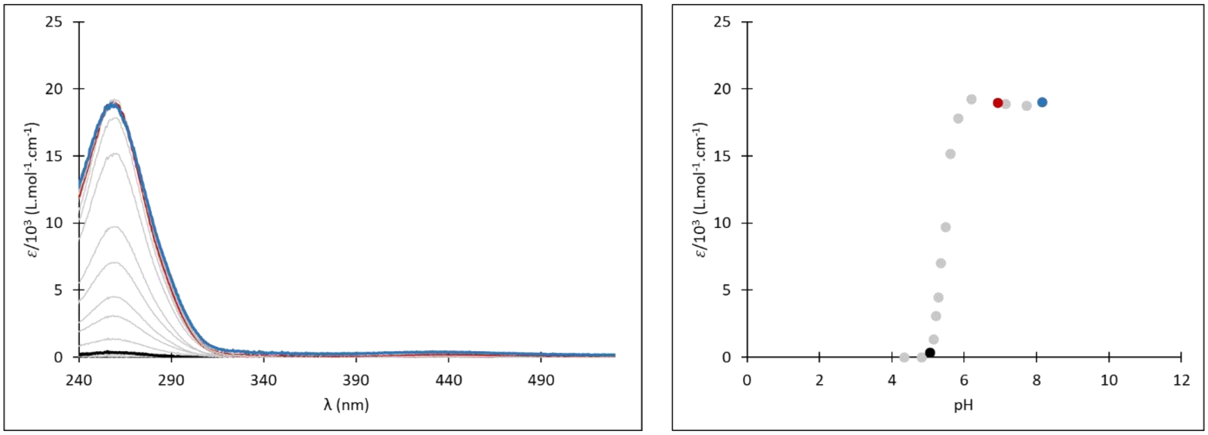

Figure S19. pH dependance of [NiCRCRR]$^{2+}$ formation. *Left: UV-vis spectra of CRCRR (56 µM) with 0.9 equiv. $NiSO_4$ in water as a function of pH. Right: absorbance evolution at 259 nm as a function of pH. Spectra and A_{262} at pH 5.3, 6.8 and 7.7 are shown in black, red and blue, respectively.*

4. Ligand titration with Ni(II)

4.1. UV-visible spectroscopy:

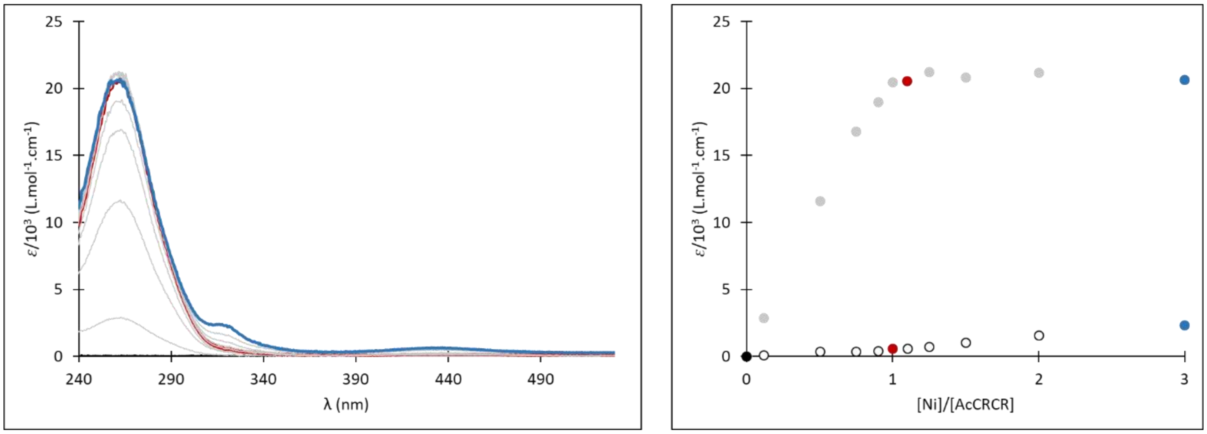

Figure S20. AcCRCR titration with Ni(II). *Left: UV-vis spectra of AcCRCR (61 µM) titrated with $NiSO_4$ in HEPES buffer (20 mM, pH 7.4, [NaCl] = 0.1 M). Spectra recorded with 0, 1 and 3 equiv. Ni(II) are shown in black, red and blue, respectively. Right: absorbance evolution at 262 nm (filled circles) and 320 nm (empty circles) as a function of Ni(II):ligand stoichiometry.*

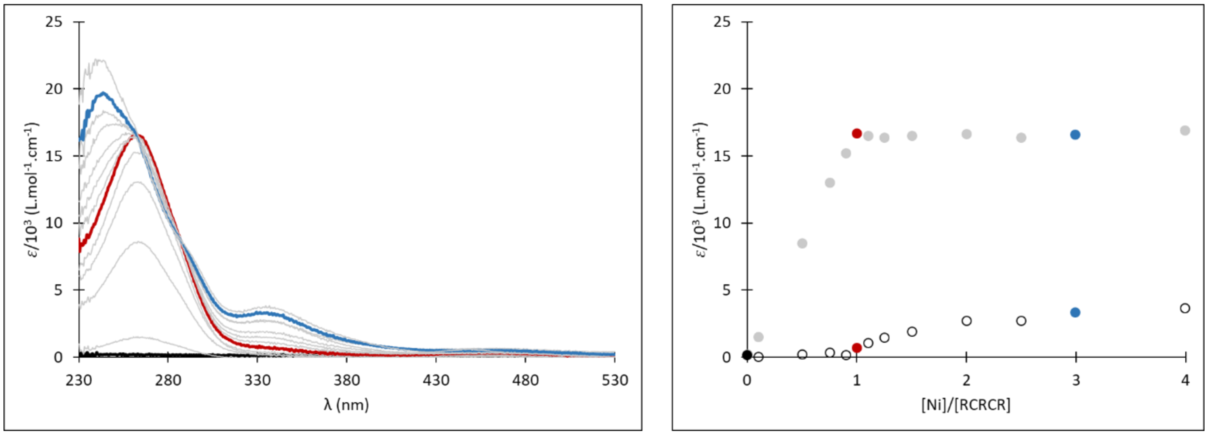

Figure S21. RCRCR titration with Ni(II). *Left: UV-vis spectra of RCRCR (35 µM) titrated with $NiSO_4$ in HEPES buffer (20 mM, pH 7.4, [NaCl] = 0.1 M). Spectra recorded with 0, 1 and 3 equiv. Ni(II) are shown in black, red and blue, respectively. Right: absorbance evolution at 262 nm (filled circles) and 335 nm (empty circles) as a function of the Ni(II):ligand stoichiometry.*

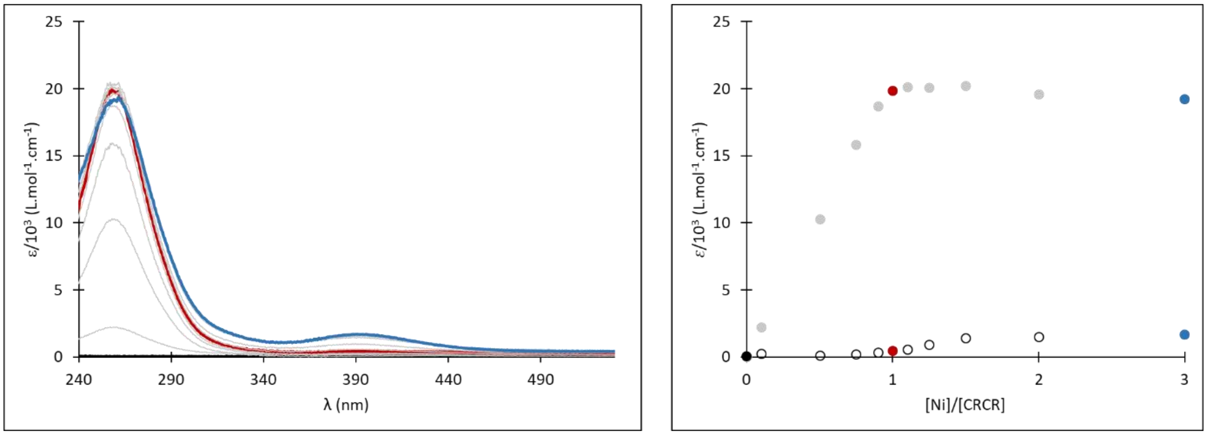

Figure S22. CRCR titration with Ni(II). *Left: UV-vis spectra of CRCR (53 µM) titrated with $NiSO_4$ in HEPES buffer (20 mM, pH 7.4, [NaCl] = 0.1 M). Spectra recorded with 0, 1 and 3 equiv. Ni(II) are shown in black, red and blue, respectively. Right: absorbance evolution at 259 nm (filled circles) and 400 nm (empty circles) as a function of the Ni(II):ligand stoichiometry.*

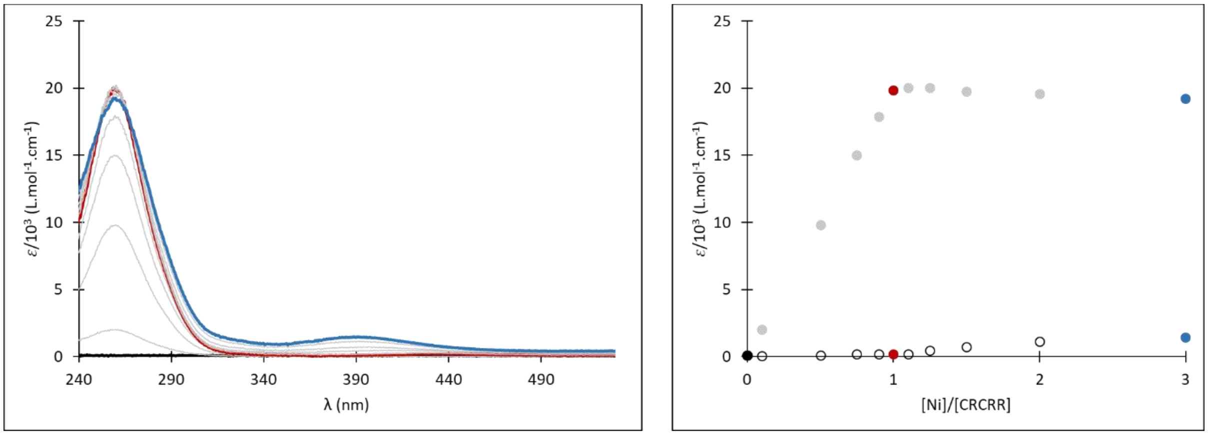

Figure S23. CRCRR titration with Ni(II). *Left: UV-vis spectra of CRCRR (53 µM) titrated with $NiSO_4$ in HEPES buffer (20 mM, pH 7.4, [NaCl] = 0.1 M). Spectra recorded with 0, 1 and 3 equiv. Ni(II) are shown in black, red and blue, respectively. Right: absorbance evolution at 259 nm (filled circles) and 400 nm (empty circles) as a function of the Ni(II):ligand stoichiometry.*

4.2. ESI-MS

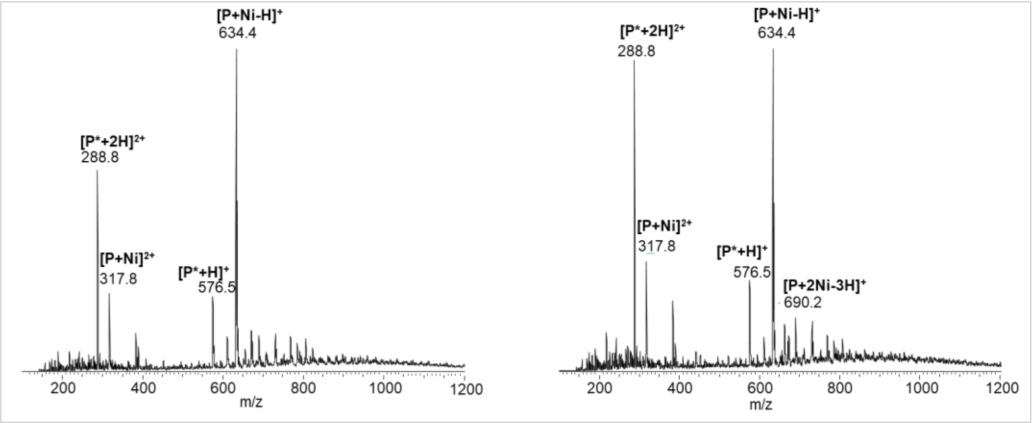

Figure S24. Mass spectra (ESI-MS, positive mode) of the neutral ligand P = [AcCRCR] (0.150 mM), with 0.9 equiv. Ni(II) (left) or 1.5 equiv. Ni(II) (right) in [AcO- NH_4^+] (20 mM, pH 6.9). P* corresponds to the oxidized peptide forming a disulfide bridge.

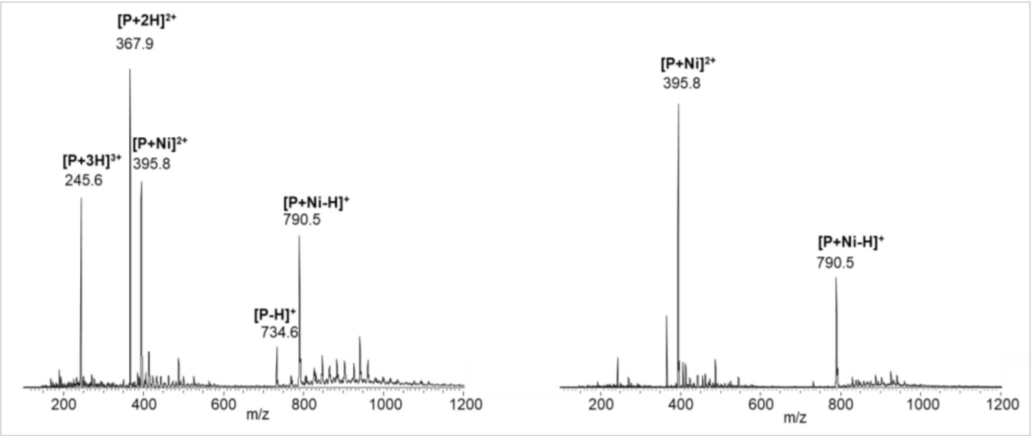

Figure S25. Mass spectra (ESI-MS, positive mode) of the neutral ligand P = [AcRCRCR] (0.150 mM), with 0.9 equiv. Ni(II) (left) or 1.5 equiv. Ni(II) (right) in [AcO^- NH_4^+] (20 mM, pH 6.9).

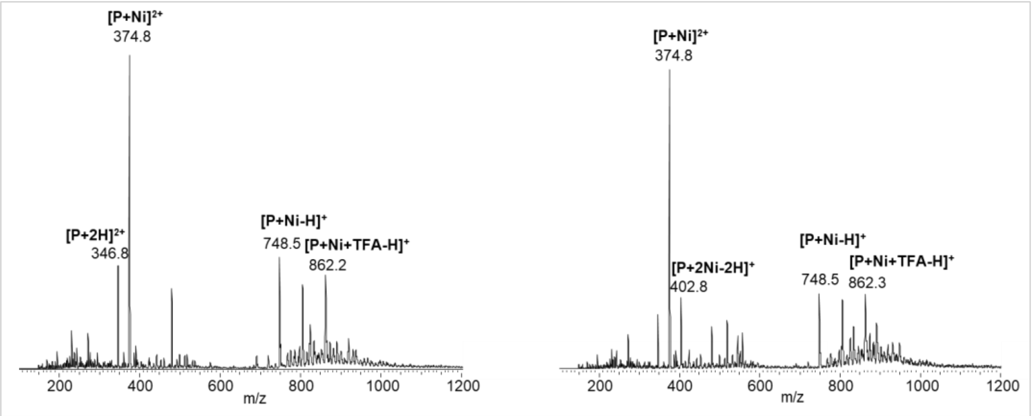

Figure S26. Mass spectra (ESI-MS, positive mode) of the neutral ligand P = [RCRCR] (0.150 mM), with 0.9 equiv. Ni(II) (left) or 1.5 equiv. Ni(II) (right) in [AcO^- NH_4^+] (20 mM, pH 6.9).

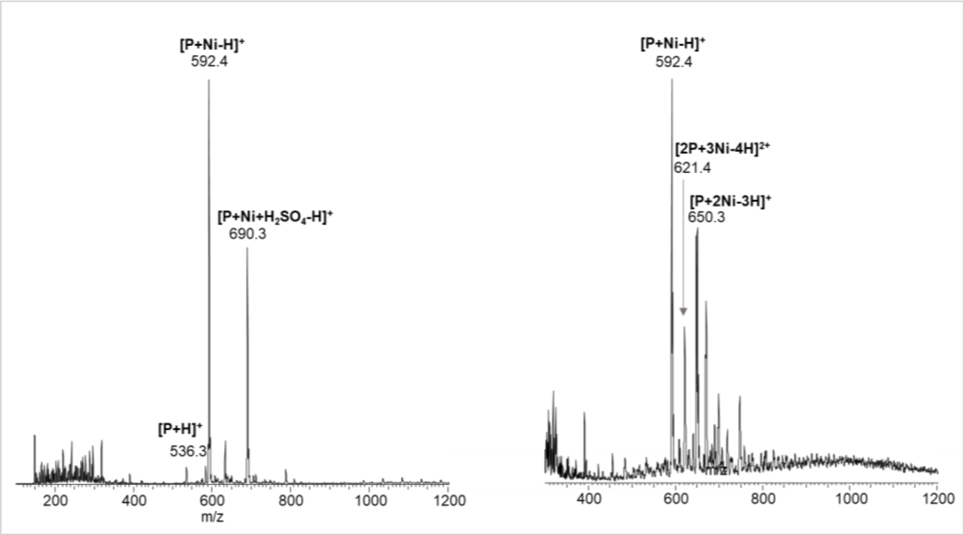

Figure S27. Mass spectra (ESI-MS, positive mode) of the neutral ligand P = [CRCR] (1.1 mM), with 0.9 equiv. Ni(II) (left) or 3 equiv. Ni(II) (right) in [AcO$^-$ NH$_4^+$] (20 mM, pH 6.9)

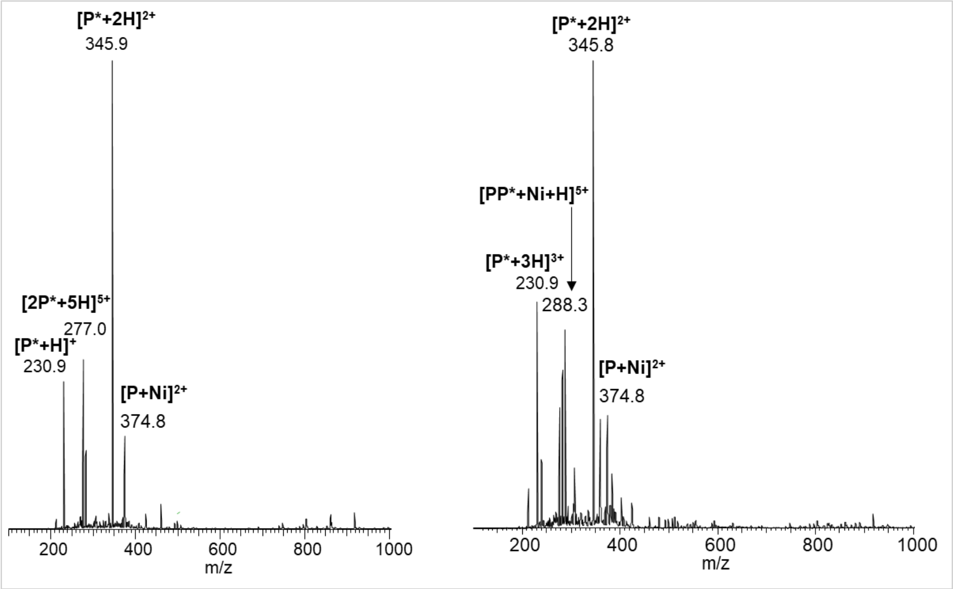

Figure S28. Mass spectra (ESI-MS, positive mode) of the neutral ligand P = [CRCRR] (0.292 mM), with 1.5 equiv. Ni(II) (left) or 3 equiv. Ni(II) (right) in [AcO$^-$ NH$_4^+$] (20 mM, pH 6.9). P* corresponds to the oxidized peptide forming a disulfide bridge.

5. Electrochemistry

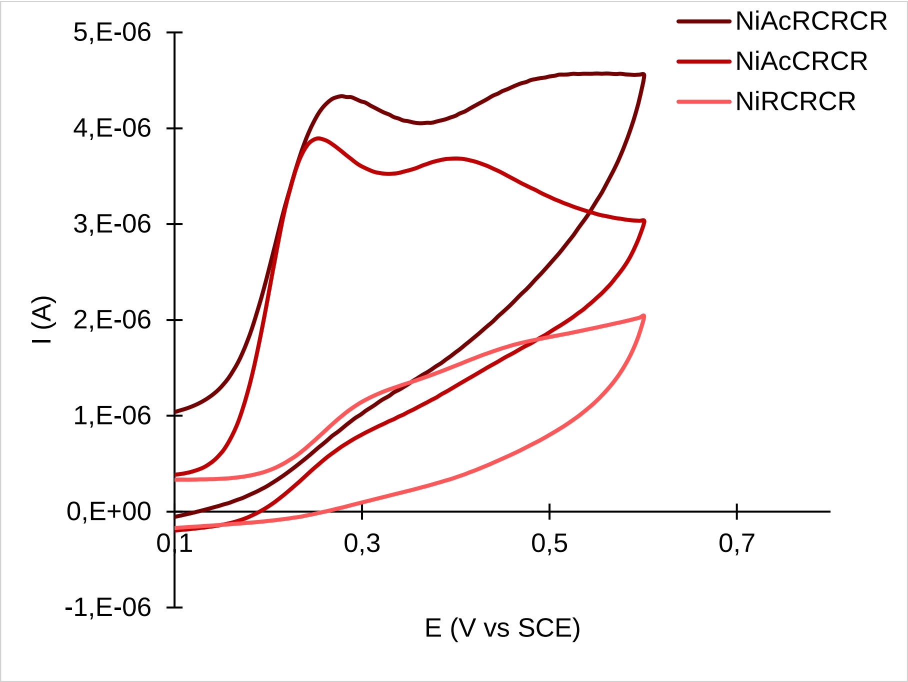

Figure S29. CV curves recorded at 50 mV s^{-1} of solutions of [NiAcCRCR]^{0}, [NiAcRCRCR]$^{+}$ and [NiRCRCR]$^{2+}$ at 400 µM in HEPES buffer (20 mM, pH 7.4, [NaCl] = 0.1 M). Working electrode: glassy carbon electrode. Reference electrode: SCE.

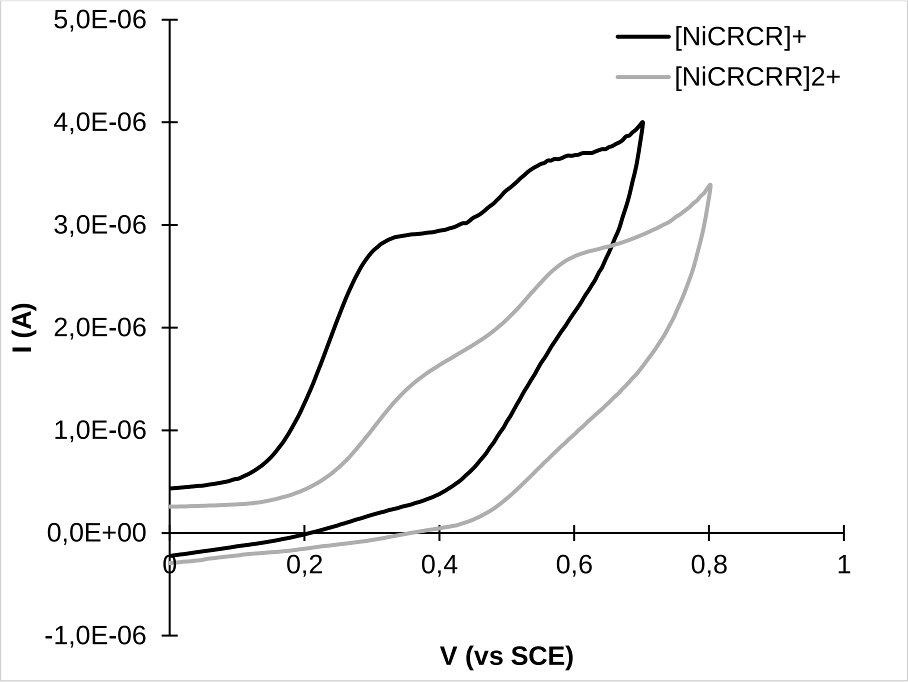

Figure S30. CV curves recorded at 50 mV s^{-1} of solutions of [NiCRCR]$^{+}$ and [NiCRCRR]$^{2+}$ at 400 µM in HEPES buffer (20 mM, pH 7.4, [NaCl] = 0.1 M). Working electrode: glassy carbon electrode. Reference electrode: SCE.

6. Catalytic activity of the complexes

Table S1. Catalytic parameters of ATCUN-like Ni(II) complexes. *% of degradation after one second of reaction with the ratio [$O_2^{\circ-}$]/[complex] described thereafter, and TONs calculated accordingly.*

Entry	Complex	% of degradation	[$O_2^{\circ-}$]/[complex]	TON[(a)]
3	[NiAcCRCR]^0	0	18	18
4	[NiAcRCRCR]$^+$	38	21	35
5	[NiRCRCR]$^{2+}$	8	18	20
8	[NiCRCRR]$^{2+}$	23	18	24

[a] *One TON corresponds to one mole of substrate consumed by one mole of complex*

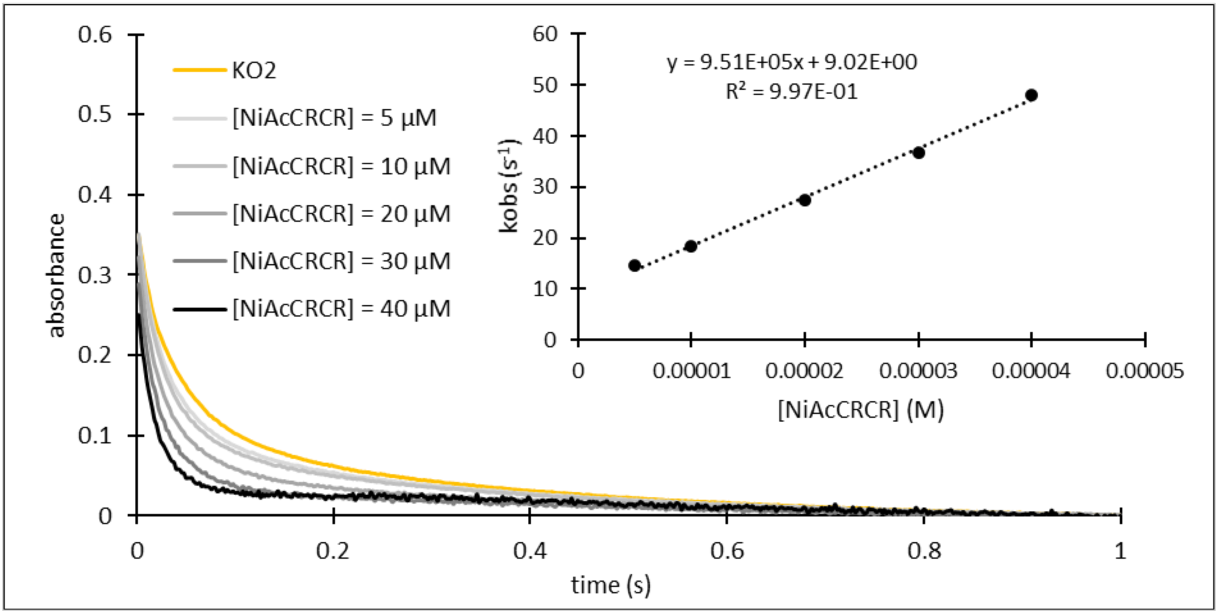

Figure S31. Catalytic activity of [NiAcCRCR]^0. *$O_2^{\bullet-}$ dismutation ([$O_2^{\bullet-}$] = 735 µM) was followed by UV/Vis at 250 nm (ε = 2,686 L.mol^{-1}.cm^{-1}) in the absence (yellow) or presence of various concentrations of complex (grey to black), as a function of time. Each trace corresponds to the mean of 4 to 9 injections. Inset: Linear regression (k_{obs}=f([complex]) with the slope corresponding to the k_{cat} value.*

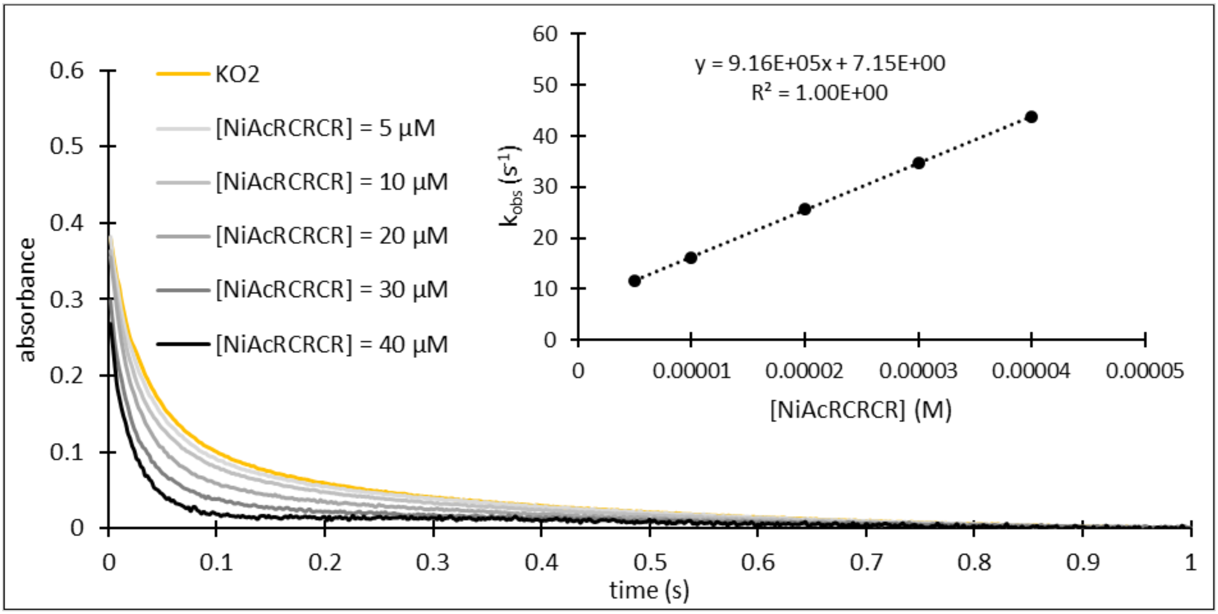

Figure S32. Catalytic activity of [NiAcRCRCR]$^{+}$. *$O_2^{\bullet-}$ dismutation ($[O_2^{\bullet-}]$ = 843 µM) was followed by UV/Vis at 250 nm (ε = 2,686 L.mol-^{1}.cm^{-1}) in the absence (yellow) or presence of various concentrations of complex (grey to black), as a function of time. Each trace corresponds to the mean of 4 to 9 injections. Inset: Linear regression (k_{obs}=f([complex]) with the slope corresponding to the k_{cat} value.*

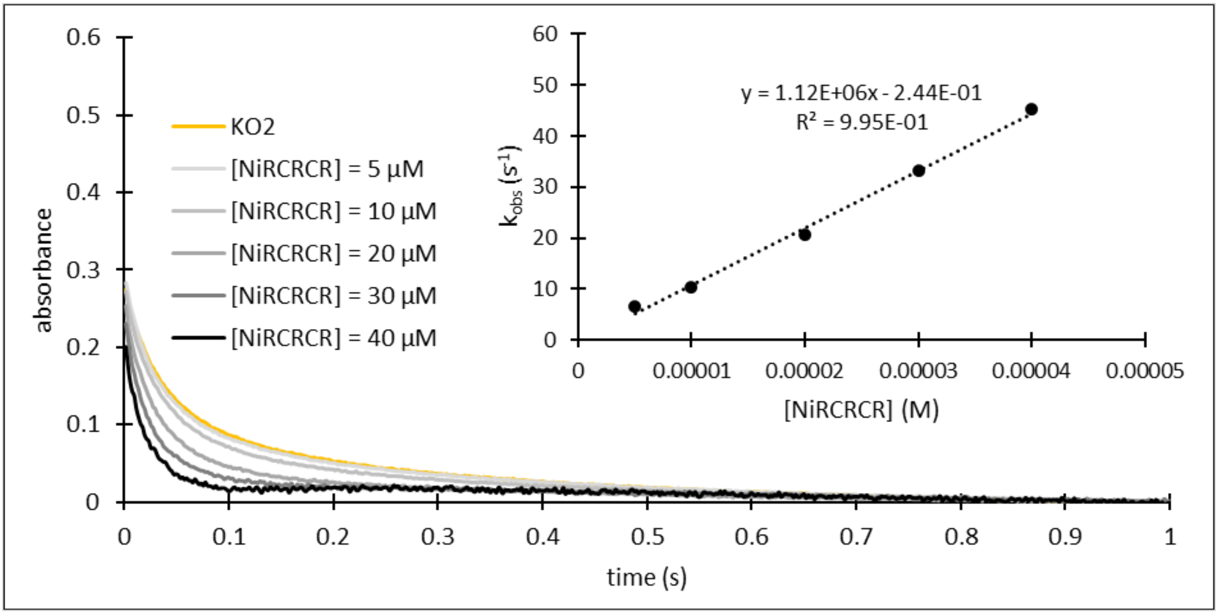

Figure S33. Catalytic activity of [NiRCRCR]$^{2+}$. *$O_2^{\bullet-}$ dismutation ($[O_2^{\bullet-}]$ = 732 µM) was followed by UV/Vis at 250 nm (ε = 2,686 L.mol-^{1}.cm^{-1}) in the absence (yellow) or presence of various concentrations of complex (grey to black), as a function of time. Each trace corresponds to the mean of 4 to 9 injections. Inset: Linear regression (k_{obs}=f([complex]) with the slope corresponding to the k_{cat} value.*

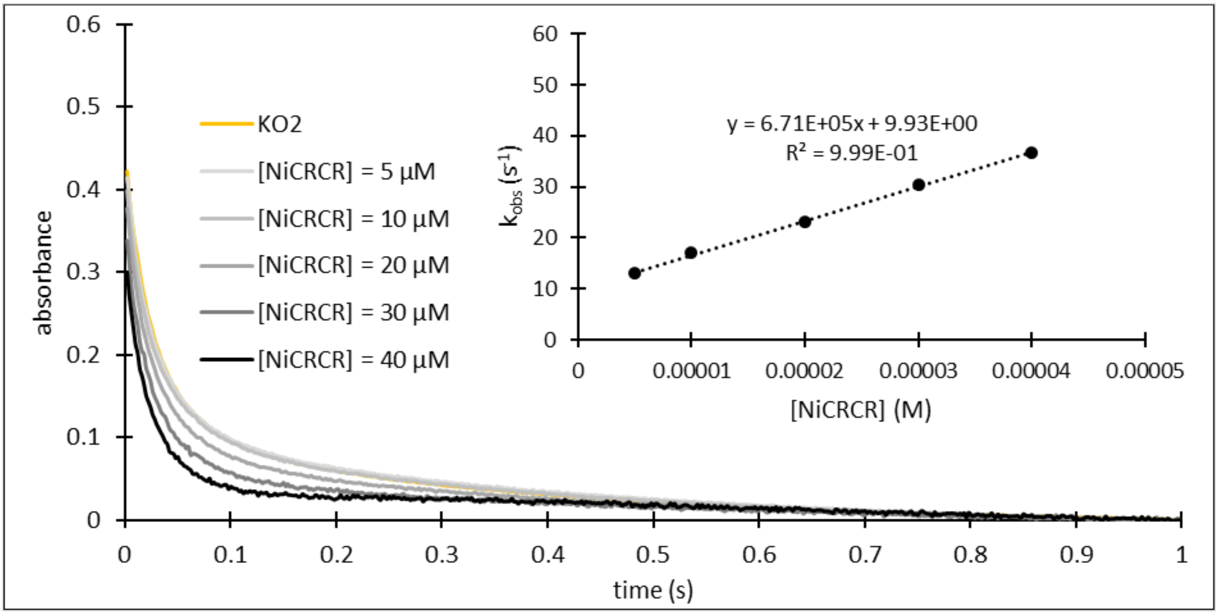

***Figure S34. Catalytic activity of [NiCRCR]*$^{+}$. $O_2^{\bullet -}$ *dismutation* ($[O_2^{\bullet -}]$ = 855 μM) *was followed by UV/Vis at 250 nm (ε = 2,686 L.mol-^{1}.cm^{-1}) in the absence (yellow) or presence of various concentrations of complex (grey to black), as a function of time. Each trace corresponds to the mean of 4 to 9 injections. Inset: Linear regression (k_{obs}=f([complex]) with the slope corresponding to the k_{cat} value.*

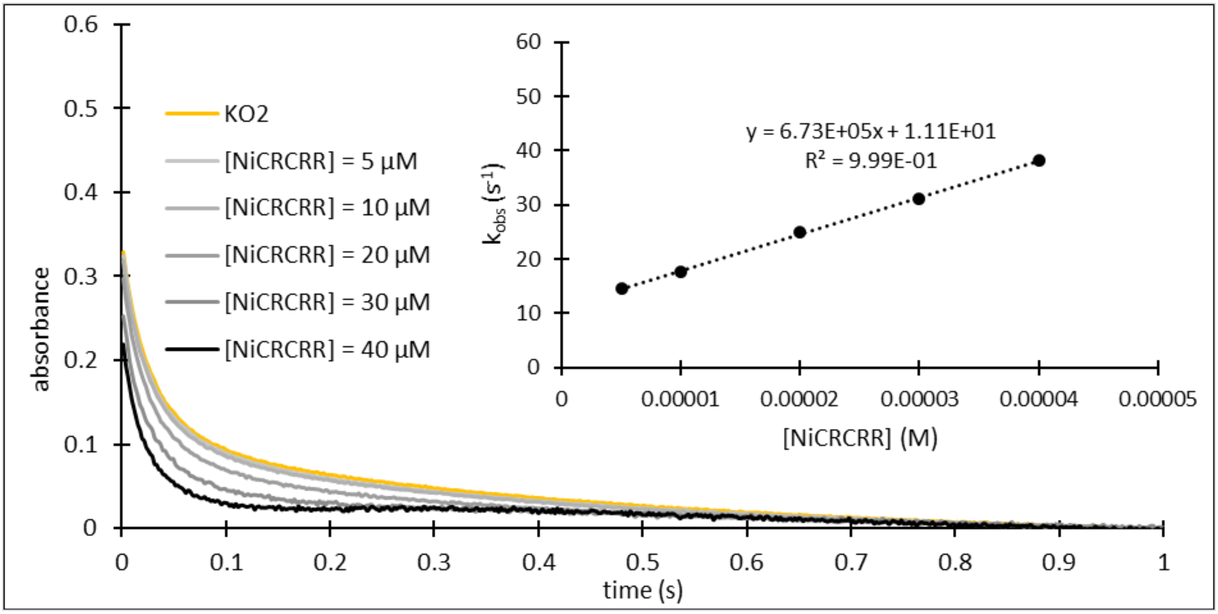

***Figure S35. Catalytic activity of [NiCRCRR]*$^{2+}$. $O_2^{\bullet -}$ *dismutation* ($[O_2^{\bullet -}]$ = 748 μM) *was followed by UV/Vis at 250 nm (ε = 2,686 L.mol-^{1}.cm^{-1}) in the absence (yellow) or presence of various concentrations of complex (grey to black), as a function of time. Each trace corresponds to the mean of 4 to 9 injections. Inset: Linear regression (k_{obs}=f([complex]) with the slope corresponding to the k_{cat} value.*

7. ¹H NMR characterization of [NiCRCR]⁺

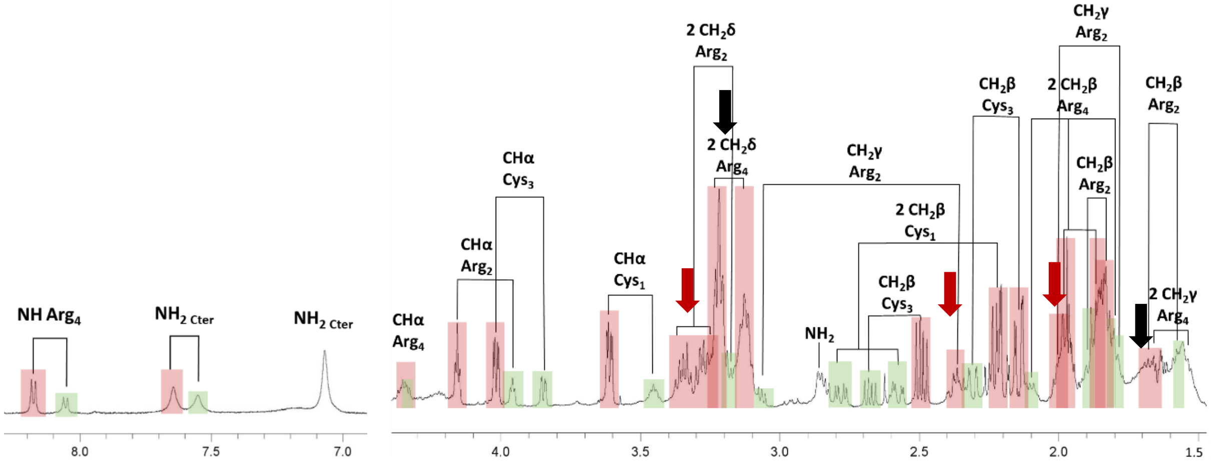

Figure S36. ***¹H NMR spectrum of [NiCRCR]⁺ (1.32 mM) at pH 8.1 in H_2O/D_2O (90 : 10) at 298 K.*** *From 8.5 to 7 ppm: watergate pulse sequence. From 5 to 1.5 ppm: presaturation. Signals of the complex in the S-coordination are highlighted in red, signals of the N-coordination in green. Red arrows underline the central Arg_2 protons, which are shifted downfield with respect to Arg_4 (black arrows) or Arg in peptides with random-coil structures: in the major S-coordination, $H^{\gamma}(Arg_2)$ 2.36/1.95 ppm ; H^{γ} (Arg_4) 1,65 ppm and $H^{\delta}(Arg_2)$ 3.35/3.28 ppm ; H^{δ} (Arg_4) 3.20/3.15 ppm.*
